# Divergent Amplification of Y-Linked Dosage-Sensitive Genes Triggers Regulatory Mismatch Underlying Cattle–Yak Male Sterility

**DOI:** 10.3390/biom16030471

**Published:** 2026-03-21

**Authors:** Yu Wang, Yulin Chen, Zhenlin Zhu, Xiaofei Zeng, Wei Ha, Longwei Su, Lian Hu, Yili Liu, Biao Li, Juan Loor, Mingfeng Jiang

**Affiliations:** 1 Key Laboratory of Qinghai-Tibetan Plateau Animal Genetic Resource Reservation, College of Animal Science and Veterinary Medicine, Southwest Minzu University, Chengdu 610041, China; wangyu@stu.swun.edu.cn (Y.W.);; 2 Agricultural Genomics Institute at Shenzhen, Chinese Academy of Agricultural Sciences, Shenzhen 518120, China; 3 Department of Animal Sciences, Division of Nutritional Sciences, University of Illinois, Urbana, IL 61801, USA

**Keywords:** reproductive isolation, spermatogenic arrest, dosage imbalance, cis-trans regulatory theory

## Abstract

As the hybrid offspring of cattle and yak, cattle–yaks suffer from male sterility, manifesting as cascading spermatogenic failure. Despite the Y chromosome’s pivotal role in spermatogenesis, the absence of a high-quality yak Y assembly has long impeded mechanistic understandings from this perspective. Here, a near-complete 42.4 Mb yak Y chromosome is constructed through a multi-stage assembly strategy that integrates de novo assembly with pangenome graph construction and Hi-C guided refinement. By developing a rigorously standardized gene annotation pipeline for precise cross-species comparison, we find that yaks have undergone a greater expansion of Y-linked ampliconic genes than cattle. Integrating this ampliconic landscape with short-read and full-length transcriptomics further demonstrates that yaks exhibit a drastic 2-to-4-fold increase in transcriptionally active copies of spermatogenesis-related ampliconic genes (including TSPY1, ZNF280BY, HSFY and PRAMEY) relative to cattle. Given negligible homology outside the pseudoautosomal region and conservation of key meiotic proteins, we propose a ‘cis-trans regulatory mismatch’ model driven by divergent Y-linked amplification as a working hypothesis to explain the primary genetic mechanism of cattle–yak male sterility. Together, these findings offer critical insights for addressing cattle–yak male sterility and establish the Y chromosome as an active driver of reproductive isolation beyond its traditional degenerate characterization.

## 1. Introduction

Over tens of millions of years, speciation has driven the diversification of life [1,2], whereas in agricultural history, distant hybridization has been extensively exploited to enhance the performance of crops [3,4,5] and livestock [6,7,8]. Although genetically closely related species can occasionally hybridize to yield viable offspring, these progeny frequently exhibit sex-biased hybrid dysfunction [9], manifesting as male sterility [10], inherently a crucial form of post-zygotic reproductive isolation conforming to Haldane’s rule where the heterogametic sex (XY) is sterile [11]. Far from being merely a failure of reproductive isolation, hybridization is also recognized as an evolutionary mechanism that facilitates speciation [12], adaptive evolution [13,14], and species persistence [15,16]. Overcoming hybrid sterility unlocks substantial economic value by retaining heterosis [17] while simultaneously revealing the genetic basis of speciation. Therefore, elucidating the underlying mechanisms through the proposal of theoretical frameworks [18], culminating in models like the regulatory theory of sex chromosome evolution [19], and applying these insights to resolve hybrid sterility in agriculturally important interspecies crosses [20] have long attracted widespread attention.

Originating from the Qinghai–Tibet Plateau, a vast region regarded as the world’s “Third Pole” [21], the cattle–yak (*Bos taurus* × *Bos grunniens*) [22] represents an ideal system to investigate post-zygotic reproductive isolation as it combines the environmental adaptability of this region’s flagship species, the yak, with the high productivity of cattle but is ultimately severely constrained by hybrid male sterility (HMS) [10]. This sterility results from the cumulative effect of a cascading spermatogenic failure characterized by at least three successive defects: (i) a severe depletion of the spermatogonial stem cell (SSC) pool, (ii) a subsequent blockage in the differentiation of spermatogonia, and (iii) a final pachytene arrest of spermatocytes [23,24,25]. Extensive omics investigations have been conducted to identify and characterize the factors driving these defects at the genomic [25], transcriptomic [26,27] (single-cell [23]), translatomic [28], proteomic [29,30] and epigenetic [31] levels, while the accurate resolution of such cascading spermatogenic failure is continuously limited by the lack of a high-quality Y chromosome assembly as the Y chromosome contains two pivotal regions closely linked to spermatogenesis: the Pseudoautosomal Region (PAR) and the Male-Specific Region of the Y (MSY), where PAR is essential to X-Y pairing, meiotic recombination, and synapsis efficiency, while MSY is critical for resolving the long-standing inaccuracy in quantifying [32,33] Y-linked dosage-sensitive spermatogenic genes [34] (e.g., TSPY, HSFY, and ZNF280BY [35]) that regulate SSC homeostasis [36,37] and interrogating the repetitive landscapes that underlie meiotic sex chromosome inactivation (MSCI).

While numerous genome assemblies have been released, a complete yak Y chromosome remains unavailable. Current assemblies are either female-derived [38], highly fragmented [39], or incomplete, covering only 26.4 Mb [40] to 35.5 Mb [41], all shorter than the estimated 43.1 Mb derived from cytogenetic evidence [42], a value also consistent with our 42.4 Mb Y-chromosome assembly presented in this study. Despite technical advances in long-read sequencing [43] and assembly algorithms [44], the immense resources required for complete Y-chromosome assembly [35] remain a barrier for most species. Therefore, incrementally accumulating sufficient data by integrating publicly available data with newly generated sequencing data constitutes a practical and cost-effective strategy for satisfying the substantial data requirements of Y chromosome assembly [45]. Furthermore, the lack of recombination and strict paternal inheritance of the Y chromosome drive the accumulation and fixation of structural variants, a hallmark of which is the extensive copy-number variation across different paternal lineages [46,47]. To identify conserved intraspecific features for interspecific comparison, the availability of multiple assemblies makes it feasible to construct a pan-chromosome graph [48] that incorporates diverse haplotypes and allows for explicit distinction of shared paths (conserved features) from lineage-specific alterations.

Here, we bridged this genomic gap by constructing a high-quality, structurally complete yak Y chromosome reference through a graph-based pan-genome approach, integrating our independent long-read and Hi-C data with diverse public datasets. The comparative genomic analysis with *Bos taurus*, built upon a complete resolution of their complex ampliconic structure with support from short-read and full-length transcriptomic evidence, revealed a substantial lineage-specific expansion of ampliconic spermatogenic genes in yak, with transcriptionally active copy numbers exceeding those in cattle by 2- to 4-fold. By integrating these insights into severe gene dosage imbalance with the concrete phenotype of male sterility in the cattle–yak within the framework of the latest regulatory theory, we elucidated the active role of the Y chromosome in cis-trans regulatory mismatch, which is manifested as Y-linked dosage-sensitive gene amplification. This study provides the definitive reference and comprehensive annotation for the yak Y chromosome, resolves persistent ambiguities about Y-linked ampliconic gene dosage, and establishes a mechanistic framework that defines the active role of the Y chromosome in speciation and explains cattle–yak male sterility through cis-trans regulatory mismatch.

## 2. Materials and Methods

### 2.1. Sample Collection

To lay the foundation for the genetic dissection of supernumerary thoracolumbar vertebrae in yak (*Bos grunniens*), we generated a de novo, chromosome-level genome assembly. This initiative was driven by the absence of any publicly available reference genome of chromosome-level quality at the project’s outset. Accordingly, a male yak with a vertebral formula of 15 thoracic and 5 lumbar (T15L5) was selected for sequencing. Samples were collected from this healthy male Maiwa yak at a commercial abattoir (the Hongyuan New Hope Yak Industry Co., Ltd., Hongyuan County, Aba Tibetan and Qiang Autonomous Prefecture, Sichuan Province, China). Small pieces of muscle tissue were cut with sterile scissors. The muscle tissues were divided into labeled 1.5 mL cryotubes and immediately put into liquid nitrogen. All animal work was reviewed and approved by Southwest Minzu University (Chengdu, Sichuan, China) Institutional Animal Care and Use Committee (IACUC).

### 2.2. Library Construction and Sequencing

Genomic DNA of the collected muscle tissues was extracted and used to construct two short-read libraries (one for polishing, whose reads were termed P-reads, and one for Hi-C, whose reads were termed H-reads) for next-generation sequencing and a long-read library for third-generation sequencing (one for assembly, whose reads were termed A-reads). The Length distribution of A-reads was presented in Figure S3.

### 2.3. Assembly of Y Chromosome

Subreads were assembled with Canu (v2.2) (Supplementary Note S1) [49]. The contigs generated from Canu were polished with NextPolish (v1.4.1) [50] using both A-reads and P-reads. The polished contigs were then scaffolded using HapHiC [51] with mapped H-reads. The scaffolds were visualized using Juicebox and adjusted manually. The whole assembly remains under study and has not yet been published. The contig-level assembly obtained here was termed Maiwa_STV1.0_C (STV: Supernumerary Thoracolumbar Vertebrae).

Next, we constructed a Y chromosome graph (Supplementary Note S2) using minigraph [48], a tool chosen for its high efficiency and scalability in iteratively incorporating additional assemblies [52]. To preclude the introduction of potential errors arising from scaffolding, our selection criteria were restricted to assemblies published at the contig level. Accordingly, we selected all available male yak genomes that met two criteria: (1) the availability of a contig-level assembly, and (2) a contig N50 greater than 30 Mb. This filtering process yielded six qualifying assemblies: our de novo assembly (Maiwa_STV1.0_C) and five others (DYXZ92, DYPK16, DYQH13, DYXJ17, and DYXZ30) (Supplementary Data S1). Among these, the Y chromosome assembly of DYXZ92 (DYXZ92_Y) was selected as the backbone for the graph due to its highest contig N50.

The contigs of each sample were mapped to the pan-chromosome graph to obtain the depth of each branch in the graph. The branches with deeper depth were preferred. And all complex branches were inspected manually (A demo of complex branches was in Supplementary Figure S3). Finally, a sequence was exported from the graph with gfatools (0.5-r292-dirty) (https://github.com/lh3/gfatools (accessed on 6 June 2024)) and termed DYY_0.5. The PAR region was missing in both DYXZ92_Y and DYY_0.5, but we found this region in our Maiwa_STV1.0_C, while we visualized our scaffolds using Juicebox [53]. The Juicebox Hi-C Contact Map of our Maiwa_STV1.0_C showed that there was a region that had high-intensity contacts with both X and Y chromosomes (Supplementary Figure S4). Further observation revealed that this region was a completely assembled contig tig00003811_np12 (PAR_Contig). This contig was aligned to DYY_0.5 and found that they overlapped. Therefore, DYY_0.5 and the PAR_Contig were merged together to generate DYY_1.0, which was the final version used for downstream analysis. The overall assembly pipeline was presented in Supplementary Figure S5.

### 2.4. Structure Resolution of Y Chromosome

The boundary of the pseudoautosomal region (PAR) on our Y chromosome assembly was determined by direct alignment of the X and Y chromosome sequences, which was similar to the previously described method [54]. First, our PAR_Contig was aligned against the DYXZ92 X chromosome (DYXZ92_X) using Mashmap (3.1.3) to estimate where sequence homology terminated. Subsequently, we used the EMBOSS water pairwise sequence alignment tool provided by the European Bioinformatics Institute (EMBL-EBI) [55] to perform the alignment with base-level precision. This alignment pinpointed the exact coordinate where sequence similarity decreased dramatically (Supplementary Figure S6A), which defined the putative PAR boundary. To validate this boundary, we then mapped sequencing reads from multiple female yaks (DYNP09 and BosGru_PB_v1.0) against the reference genome (DYXZ92, with its original Y replaced by our DYY_1.0). Telomere repeat sequences were identified using seqtk (v1.4).

### 2.5. Repeat Elements Annotation

To ensure a direct and unbiased comparison, we annotated transposable elements (TEs) in both our DYY_1.0 assembly and the ARS_UCD_2.0_Y reference using an identical workflow. This step was critical to mitigate potential biases, as TE annotation results are known to vary between different software tools. The annotation was performed with the Earl Grey pipeline [56], utilizing the Mammalia branch of the Dfam 3.9 database (March 2025) for TE identification and classification.

### 2.6. Structural and Functional Annotation of Genes

Our gene annotation pipeline for the DYY_1.0 assembly integrated both lift-over and de novo approaches to generate a comprehensive and high-quality gene set. The initial lift-over step using Liftoff [57] aimed to directly map high-confidence annotated genes from the cattle (*Bos taurus*) Y chromosome onto the yak (*Bos grunniens*) Y chromosome, serving as the first round of gene annotation for the yak. To capture potentially novel or divergent genes not present in the cattle reference, we conducted a parallel de novo gene prediction using BRAKER3 [58]. The outputs from both Liftoff and BRAKER3 were subsequently merged. Following the finalization of gene structures, functional annotation was performed by assigning protein functions based on homology searches against the Swiss-Prot database [59]. Further details were provided in Supplementary Note S3.

### 2.7. Assessing the Transcriptional Activity of Ampliconic Genes

To ensure alignment accuracy and minimize potential mapping biases, raw reads were mapped against the entire genome rather than restricting the alignment solely to the Y chromosome. Short-read RNA-seq data were aligned using HISAT2 (v2.2.1) [60]. Subsequently, SAMtools (1.9) was used to sort and index the resulting files, after which reads mapping specifically to the Y chromosome were extracted for further analysis. Gene-level quantification was performed using featureCounts (2.0.1) [61] to calculate read counts for each gene. For long-read full-length transcriptome data, alignment was carried out using minimap2 [62], followed by the same downstream processing pipeline as the short-read data. Detailed information regarding tissue types and data sources is provided in Supplementary Data S2.

The copy numbers of eight key ampliconic genes were evaluated using three metrics: Annotation-Supported Copies (ASC), Transcript-Supported Copies (TSC) and high-confidence Transcript-Supported Copies (hTSC). These metrics assessed the genes at the genomic and transcriptomic levels, respectively. At the genomic level, our approach moved beyond simple sequence alignment. Instead of using BLAST with a canonical sequence, which would identify a larger set of copies based on similarity alone, we narrowly defined ASCs as copies identified by rigorous gene prediction tools that possess a complete and accurate gene structure. Copies identified as ASCs were therefore more likely to have retained their biological function compared to those identified by BLAST (2.11.0+) based on sequence homology alone. In essence, ASC represents the gene copy number determined through the gene annotation pipeline described above.

TSCs were then defined as the subset of ASCs to which transcriptomic reads could be successfully mapped, providing direct evidence of transcription. Therefore, TSCs represented the copies being transcriptionally active. However, due to the short length of second-generation reads, mapping to highly similar ampliconic copies often involves multi-mapping (randomness in alignment), which artificially inflates the number of identified active copies (TSCs). In contrast, third-generation long-read data provides superior mapping resolution and specificity, allowing reads to be precisely assigned to their corresponding copies. Thus, compared to TSCs identified using short reads, those identified using long reads provide a much more accurate estimation of genuinely transcriptionally active copies, and are referred to as high-confidence TSCs (hTSCs) herein.

Furthermore, since transcriptional activity alone does not guarantee the production of functional proteins, it is crucial to verify whether the protein sequences encoded by TSCs retain essential functional motifs. Consequently, we performed protein domain analysis. Specifically for TSPY1, which exhibited the most dramatic copy number expansion in the yak genome, we utilized InterProScan [63] 5.73–104.0 to verify the presence of the conserved NAP domain in the protein sequence of each predicted copy.

### 2.8. Evaluation of Assembly Completeness and Continuity

To make our assembly comparable to these cytogenetic measurements, we calculated its relative proportion within the genome. This was done by computing the ratio of the DYY_1.0 assembly length to the total size of the reference genome (BosGru3.1, after substituting our DYY_1.0 for the original Y chromosome). We chose BosGru3.1 primarily because it is widely used and currently serves as the representative yak assembly in both the NCBI and Ensembl databases. While there are other assemblies available, such as NRCY_Bgru_v1 and NWIPB_DYAK_1.0, and NRCY_Bgru_v1 has the highest contiguity, its total genome size (2.8 Gb) is nearly identical to that of BosGru3.1. Because the overall sizes of these two key assemblies are consistent, differences in autosome size between them are negligible and do not significantly affect the calculated chromosomal percentages.

Long-read sequencing data were aligned to the reference genomes using Minimap2 [62]. The sequencing depth was calculated using Mosdepth (0.3.8) [64]. Detailed information regarding the samples, data volume, and sources of the long-read sequencing data is provided in Supplementary Data S3.

### 2.9. Collinearity Analysis of Sex Chromosomes

For whole-genome dot plot visualization, alignment data was generated using two complementary tools: Mashmap [65] for rapid, large-scale comparisons, and MUMmer (4.0.0rc1) [66] for more sensitive, high-resolution alignments. The outputs from both Mosdepth and the alignment tools were subsequently visualized using custom in-house scripts.

### 2.10. Triangular Dot Plots and Distribution Plots

ModDotPlot (v0.9.1) [67] was used to perform self-comparison analysis and generate triangular dot plots. This tool employs an efficient k-mer sketching algorithm to rapidly approximate nucleotide identity, making it particularly adept at visualizing repetitive structures. Linkview2 (https://github.com/YangJianshun/LINKVIEW2 (accessed on 8 August 2025)) was used to draw distribution [68] plots of ampliconic genes.

### 2.11. Prediction of PRDM9 Binding Motif

PRDM9 is a canonical C2H2-type zinc finger (ZF) protein, in which each ZF motif is stabilized by the tetrahedral coordination of a central zinc ion (Zn^2+^) by two cysteine and two histidine residues [69]. The C2H2 zinc finger (ZF) is a modular DNA-binding domain, where a single finger typically recognizes a 3-base-pair (bp) sequence. By assembling multiple fingers into a tandem array, they can be engineered to target longer, more specific DNA sequences [70]. The high polymorphism of the PRDM9 zinc finger domain in bovids likely results in the activation of distinct meiotic recombination hotspots [71]. A computational method has been introduced to predict the DNA-binding specificity of C2H2 zinc fingers as a Position Weight Matrix (PWM). The underlying principle is to first systematically calculate the binding scores for a zinc finger against all possible DNA target sequences, and then statistically convert these scores into a probabilistic binding profile [72].

### 2.12. Assessment of the Conservation of Orthologues in Meiotic Recombination

The genes in the list were manually curated from the literature (Supplementary Note S4). Protein sequence similarity was assessed based on the ‘Target %id’ and ‘Query %id’ metrics provided by Ensembl for orthologues. First, the genes were located within the cattle genome, which was selected for its high annotation completeness. Subsequently, the ‘Orthologues’ analysis was accessed by navigating to the ‘Comparative Genomics’ section under the ‘Gene-based displays’ sidebar. The resulting page displays the orthologues identified by the Ensembl pipeline. In cases where protein sequences in Ensembl exhibited high divergence or were absent from the genome annotation, protein sequence similarity was instead assessed using NCBI data. Specifically, protein sequences from the cattle reference genome (ARS-UCD2.0) were aligned against the two yak reference genomes (BosGru3.1 and NWIPB_DYAK_1.0) using tblastn. Protein sequence similarity was evaluated based on query coverage and percent identity. Two distinct yak genome assemblies were employed, as certain genes in a particular genome can still exhibit poor assembly quality. For certain genes (e.g., RAD51, METTL16) whose reliable similarity assessments could not be obtained from either database, as Ensembl annotations indicated low similarity and subsequent NCBI alignments proved ambiguous or of low similarity, protein sequences predicted from our in-house Maiwa_STV1.0 genome were aligned against high-quality cattle orthologues using EMBOSS Needle. Overall, the data presented in the table represented the maximum sequence similarity scores obtained across all alignment strategies. This strategy of determining protein sequence similarity through multiple methods was necessary because annotated protein sequences for many poorly characterized genes were often of low quality, which could otherwise lead to an overestimation of the protein sequence divergence between the two species.

## 3. Results

### 3.1. Assembly and Assessment of a Representative Yak Y Chromosome

The final, high-quality representative Y-chromosome assembly (DYY_1.0) was derived from a manually curated pan-chromosome graph, which integrated a de novo assembly from our male supernumerary thoracolumbar-vertebra yak (Maiwa_STV1.0) with existing assemblies from five other male yaks (DYXZ92, DYPK16, DYQH13, DYXJ17 and DYXZ30), using minigraph for graph construction and Hi-C data to guide the addition of the PAR. The short-read libraries were sequenced on the Illumina Hiseq platform and generated 80.91 Gb (for polishing, P-reads) and 237.92 Gb (for Hi-C, H-reads) clean reads. The long-read library was sequenced on the PacBio Sequel II platform and generated 338.96 Gb subreads (for assembly, A-reads, Supplementary Figure S7, Supplementary Data S4). Finally, we generated a 42.4 Mb (Table 1) near-complete representative yak Y assembly (DYY_1.0), with a relative length in close agreement with the evaluations from two previous cytogenetic studies [42,73]. These two cytogenetic studies, which measured chromosome lengths on photomicrographs (1000× magnification), reported the mean relative length of the Y chromosome as 1.51 ± 0.05% (sample size = 64 male and 64 female yaks [42]) and 1.94 ± 0.07% (sample size = 20 male and 20 female yaks [73]), respectively. The proportion of the Y chromosome in the yak genome (1.49%) aligns closely with the cytogenetic evidence (1.51 ± 0.05%), supporting the completeness of our assembly (Supplementary Data S5). To validate the accuracy of these relative length comparisons, we extended the analysis to the X chromosome. We found it constitutes 4.80% of the genome, closely mirroring the 4.72% reported in the larger-scale cytogenetic study [42]. This agreement across both sex chromosomes further supports the biological completeness of the DYY_1.0 assembly.

To comprehensively evaluate the assembly quality of DYY_1.0, we aligned long-read sequencing data from both yak and cattle individuals to their respective reference genomes. First, the alignment of yak data to DYY_1.0 confirmed the structural integrity and sex-specificity of our assembly (Supplementary Figure S8). Male long-reads exhibited highly uniform and continuous coverage across the majority of the 42.4 Mb sequence, whereas female long-reads mapped almost exclusively to the Pseudoautosomal Region (PAR), validating the high purity of DYY_1.0. A comprehensive examination of read coverage across the entire Y chromosome revealed a specific region with reduced coverage near the end opposite to the PAR. When mapping reads from the yak individual M_Maiwa_STV1.0 to DYY_1.0, a narrow low-coverage region (~0.5 Mb) was observed near the 41 Mb, while coverage remained stable for other regions. In stark contrast, alignments to the *Bos taurus* reference (ARS-UCD2.0) revealed a systematic quality issue: all three examined cattle individuals (M_RGVxSIM, M_Braunvieh, and M_YAU_Btau_1.0) exhibited a ~2 Mb region with extremely low depth between 55 and 57 Mb (Supplementary Figure S9). The consistent lack of coverage across all cattle samples in this specific region suggests that the sequence near the end opposite to the PAR of the current cattle reference (ARS-UCD2.0) may not be universally shared among individuals, whereas DYY_1.0 demonstrates superior continuity and representativeness.

### 3.2. Comparison of the Composition of Yak and Cattle Y Chromosomes

The yak Y chromosome was 42.42 Mb in length, which was 17.05 Mb shorter than that of cattle (Table 1). However, the pseudoautosomal region (PAR) in yak was slightly longer at 6.84 Mb, exceeding that of cattle by 32.31 Kb. Consequently, the PAR accounted for 16.13% of the total Y chromosome length in yak, compared to 11.45% in cattle. The regions outside the PAR experienced extensive expansion in cattle. The proportion of repetitive elements on the yak Y chromosome was 57.22%, whereas the previously reported content for the cattle Y chromosome was 49.46% [35]. To ensure comparability, we applied the same repeat annotation pipeline to the cattle Y chromosome, which yielded a repeat content of 54.14%. The proportion of repetitive elements in the yak genome (BosGru3.0 whole-genome assembly) was reported to be 43.90% [40], which was over 15 percentage points lower than that of the Y chromosome, indicating a substantial enrichment of repetitive elements on the Y chromosome compared to the autosomes.

Among the annotated transposable elements on the Y chromosome (Table 1), Class I retrotransposons constituted more than 90% in both yak and cattle, followed by Class II DNA transposons (~5%) and other repeat classes (<1%). However, interspecies differences were observed in their specific proportions. The yak Y chromosome contained a 3.02 percentage point higher proportion of Class I retrotransposons and a 3.03 percentage point lower proportion of Class II DNA transposons compared to cattle. Within Class I retrotransposons, Long Interspersed Nuclear Elements (LINEs) were the most abundant type in both species. In yak, LINEs constituted 74.90% of the repeat elements, while in cattle, they accounted for a comparable 75.42%. Following LINEs, Long Terminal Repeat (LTR) elements and Short Interspersed Nuclear Elements (SINEs) represented the next most abundant transposable element (TE) classes in both species. Their relative contributions were also similar, with LTRs covering 10.69% in yak and 8.71% in cattle, and SINEs covering 9.54% in yak and 8.00% in cattle. Other elements, such as Penelope, were found to be exceptionally rare in both lineages. The most notable quantitative difference lay in the contribution of Rolling Circle (Helitron) elements. Within the Helitron superfamily, the HELITRON7_MMYO family occupied 2,003,539 bp in the cattle Y chromosome and 717,577 bp in the yak Y chromosome. The total length of Helitron accounted for 3.38% of the cattle Y chromosome, roughly double the percentage in yak (1.71%). This disparity was even more pronounced in absolute terms, with the total length of Helitron sequences being nearly 3-fold greater in cattle (2.01 Mb vs. 0.72 Mb), accompanied by a similarly dramatic increase in copy number (7255 vs. 2745).

We initiated the delineation of the PAR boundary by first aligning the PAR-containing contig (PAR_Contig) from the Maiwa_STV1.0 assembly directly to the X chromosome of the DYXZ92 reference genome. This initial analysis revealed that the sequence similarity terminated between 6.858–6.859 Mb on the PAR_Contig and 7.031–7.032 Mb on the DYXZ92 X chromosome. Next, two 5-kb windows encompassing these termination intervals (6,855,000–6,860,000 bp of PAR_Contig and 7,030,000–7,035,000 bp of DYXZ92 X) were extracted for a detailed pairwise alignment. The high sequence similarity was found to extend precisely to position 6,858,916 bp on the PAR_Contig. Immediately downstream of this site, we observed a 9-bp deletion in the PAR_Contig, followed by extensive sequence divergence characterized by numerous mismatches and InDels (Supplementary Figure S6A). This sharp drop in sequence identity demarcates the boundary of the PAR.

**Figure 1:**
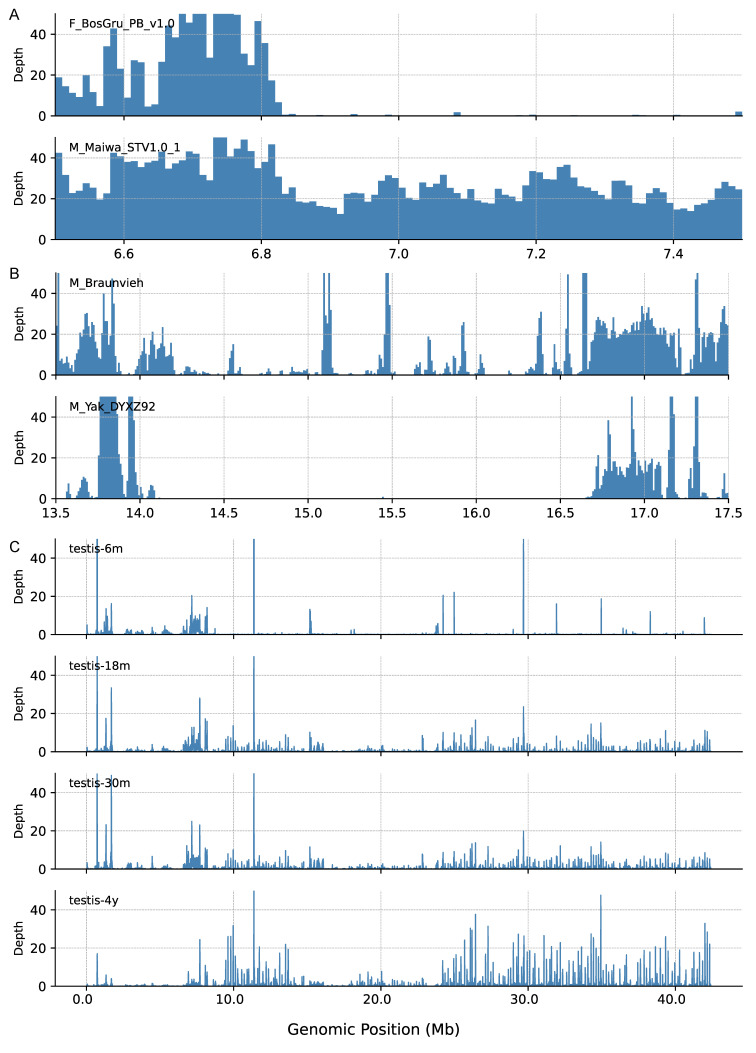
Genomic and transcriptomic read depth profiles across specific Y chromosome regions: (**A**): PAB read depth on the yak Y chromosome (DYY_1.0) for female (F_BosGru_PB_v1.0) vs. male (M_Maiwa_STV1.0_1) yaks. (**B**): Centromeric read depth in cattle, comparing male cattle (M_Braunvieh) and male yak (M_Yak_DYXZ92). (**C**): Full-length transcriptome (Iso-Seq) read depth across the yak Y chromosome (DYY_1.0) at four testicular developmental stages: 6 months (testis-6 m), 18 months (testis-18 m), 30 months (testis-30 m), and 4 years (testis-4 y).

Validation of this boundary and assessment of its conservation were conducted by mapping long-read sequencing data from two female (DYNP09 and BosGru_PB_v1.0) and one male (Maiwa_STV1.0) yaks to a modified reference genome (DYXZ92 with its Y chromosome replaced by DYY_1.0). Coverage analysis of the 6.8–7.0 Mb region on the Y chromosome showed that read coverage from female yaks terminated within this interval, whereas coverage from the male yak continued seamlessly beyond it (Figure 1A), consistent with the presence of a PAR boundary. A comparison between the two females (DYNP09 and BosGru_PB_v1.0) at the chromosome scale showed no obvious difference in the PAR termination site (Supplementary Figure S9). However, a higher-resolution analysis within a 99-kb window revealed a subtle inter-individual variation: the PAR ended at approximately 6.855–6.860 Mb in DYNP09 and at 6.825–6.830 Mb in BosGru_PB_v1.0, a difference of roughly 30 kb (Supplementary Figure S6B). Furthermore, fine-mapping in DYNP09 localized its boundary to a specific base at 6,858,509 bp (Supplementary Figure S6C), which is within 500 bp of the boundary (6,858,916 bp) identified in PAR_Contig. Our analysis demonstrates that the length of the PAR in yak is not perfectly conserved among individuals. This phenomenon of variable PAR length mirrors observations on the human Y chromosome [46]. Due to the high sequence similarity between the cattle and yak Y chromosomes, which extends to approximately 8.75 Mb, and given that the PAB in both species is located around 6.8 Mb, the genomic regions adjacent to the PAB in cattle and yak have remained relatively conserved and have not yet diverged significantly.

The telomere sequence at the PAR end was identified within the PAR_Contig as an 18.9 kb tandem repeat identical to the cattle’s CCCTAA sequence (Table 1), whereas the telomere at the q-arm and the centromere were not resolved. The centromeric region constitutes canonical heterochromatin, typically appearing as a genomic interval depleted of interspersed repeats (such as LINEs and SINEs) and genes. This characteristic pattern was clearly observable on the cattle Y chromosome, marking the successfully assembled centromere. In contrast, this specific landscape was absent in the DYY_1.0 assembly, suggesting that the centromeric region was likely not captured in the current assembly. Further investigation into the sequence divergence of the Y chromosome centromere between yak and cattle involved aligning whole-genome sequencing (WGS) data from a yak individual (DYXZ92) to the cattle ARS-UCD2.0 Y chromosome. A complete lack of coverage was observed within the annotated centromeric region (14.1–16.6 Mb), resulting in a distinct coverage gap (Figure 1B, M_Yak_DYXZ92). This absence of read mapping strongly indicated significant sequence divergence in the centromeric satellite repeats between the two species. In stark contrast, WGS data from two cattle breeds, Braunvieh and a RGVxSIM crossbreed, exhibited substantial coverage across this same region (Figure 1B, M_Braunvieh; Figure S8, M_RGVxSIM), thereby validating the integrity of the centromeric sequence in the ARS-UCD2.0 Y chromosome and confirming its cattle origin. Furthermore, we explored intra-species diversity by mapping data from two female cattle and one Yunling individual, which showed either a complete lack of coverage (Supplementary Figure S10, F_DWZxEVO and F_GIRxSIM) or only partial coverage over the centromeric sequence (Supplementary Figure S10, M_YAU_Btau_1.0). Collectively, these findings demonstrated not only a clear inter-species divergence of the Y centromere between yak and cattle but also revealed the existence of considerable sequence or structural diversity across different cattle lineages and between sexes.

### 3.3. Y-Chromosomal Landscape of Repetitive Sequences and Ampliconic Genes

**Figure 2:**
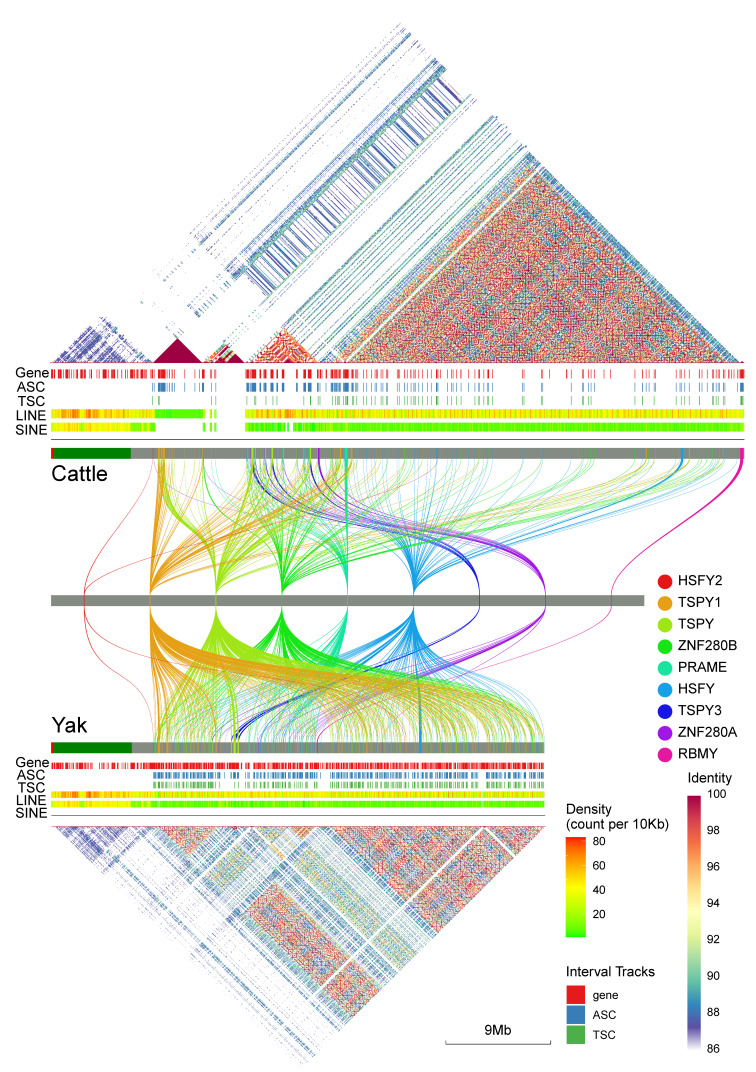
Distribution of Repeat Elements and Ampliconic Gene Families on the Cattle and Yak Y Chromosomes. (Middle) Arrangement of nine selected ampliconic genes/gene families. Cattle (**top**) and yak (**bottom**) Y chromosomes are compared against a central virtual reference, with connecting lines indicating the copy number and distribution of Annotation-Supported Copies (ASCs) in each species. (**Top** and **Bottom**) Triangular dot plots display intra-chromosomal self-similarity, where colors indicate the percentage of sequence identity. (Intermediate Tracks) Five tracks between the collinearity and dot plots show the distribution of: (i) overall genes, (ii) ASCs, (iii) Transcript-Supported Copies (TSCs), (iv) LINEs, and (v) SINEs.

The gene distribution plot in the middle of Figure 2 revealed that all analyzed ampliconic gene families’ Annotation-Supported Copies (ASCs) reside within the non-pseudoautosomal region (non-PAR), and thus did not engage in homologous recombination with the X chromosome. We specifically chose to map ASCs rather than Transcript-Supported Copies (TSCs) as ASCs provided a more comprehensive evolutionary record. The ASCs included transcriptionally silent copies and pseudogenes, which served as evolutionary footprints of past amplification [74] that illuminated the unique history of gene duplication events within each species. On the human Y chromosome, the multi-copy TSPY gene is organized as a tandem array [34]. In cattle and yak, a similar tandem array of TSPY1 was observed. However, TSPY1 had also undergone massive amplification, resulting in numerous copies that were widely distributed across the entire chromosome.

By classifying the ampliconic gene families based on their chromosome distribution relative to the rightmost ASC of PRAMEY as a reference landmark (hereafter the PRAMEY-landmark copy), we identified three distinct patterns of organization. The first group, termed ‘anteriorly restricted’, included gene family TSPY3 and ZNF280AY, whose ASCs are all located upstream of this landmark. The ZNF280AY family in the yak presented an exception, with one of its ASCs found downstream of the PRAMEY-landmark copy. The second group, designated ‘posteriorly restricted’, consisted of a family RBMY, which are found exclusively downstream of this landmark. However, their distribution pattern differed between the two species: while the RBMY copies in cattle were all clustered at the far downstream end of the Y chromosome, those in yak were concentrated in the central region of the chromosome, immediately downstream of the PRAMEY-landmark copy. Finally, a third group, categorized as ‘widely dispersed’ and including HSFY2, TSPY1, TSPY, ZNF280B and HSFY, featured ASCs that flank the landmark, spanning a significant portion of the non-PAR.

The triangular dotplots at the top and bottom of Figure 2 depicted the intra-chromosomal self-similarity of the Y chromosome. The non-PAR of the Y chromosome counteracts degeneration by amplifying key genes to create functional redundancy. Mechanistically, this process arises from large-scale segmental duplications rather than targeted, gene-by-gene copying [75]. The evolutionary history and structural outcomes of these duplications can be precisely visualized and reconstructed using dot plot analysis. The PARs of cattle and yak were both characterized by low intra-regional self-similarity, with the identity between its repetitive elements largely confined to the 86–90% range. It also shared minimal sequence identity with the rest of the Y chromosome. These features clearly delineated a distinct evolutionary trajectory for the PAR, which was shaped by meiotic recombination, setting it apart from the non-recombining (non-PAR) regions of the Y chromosome. Outside the PAR, distinct repetitive architectures were observed. The cattle non-PAR region contained four blocks of high self-similarity sequence (HS; >95% identity), visualized as red triangular regions. The yak non-PAR, in contrast, was composed of three HS blocks and one block of medium self-similarity sequence (MS; 90–95% identity).

The architecture of the cattle non-PAR differs markedly from that of the yak. In cattle, four blocks of high sequence similarity (HS blocks) cover over 95% of this region. However, the three HS blocks in the yak non-PAR covered only 85% of this region, with the remaining 15% being not part of any large, highly repetitive block. Because the Y chromosome lacks a homologous partner for meiotic pairing and self-repair, sequence amplification is a critical mechanism for protecting essential sequences from physical loss and disruptive mutations. Therefore, the higher coverage of HS blocks in cattle might suggest that its Y chromosome has reached a more functionally stable evolutionary state, where the protection of essential sequences is more firmly established. The degree of sequence identity between these blocks also differed between the two species. In cattle, the four HS blocks shared low similarity with one another, with most inter-block identities falling below 85% or within the 85–90% range. In yaks, however, a higher degree of homology was observed between blocks; specifically, a high identity was observed between the first and third HS blocks, and the MS block also shared a medium level of identity (90–95%) with them. This observation suggested that the duplicated sequences at different loci in cattle had undergone more significant divergence, whereas those in yak have not yet diverged as deeply.

### 3.4. Drastic Divergence in Ampliconic Gene Dosage Between Yak and Cattle

**Figure 3:**
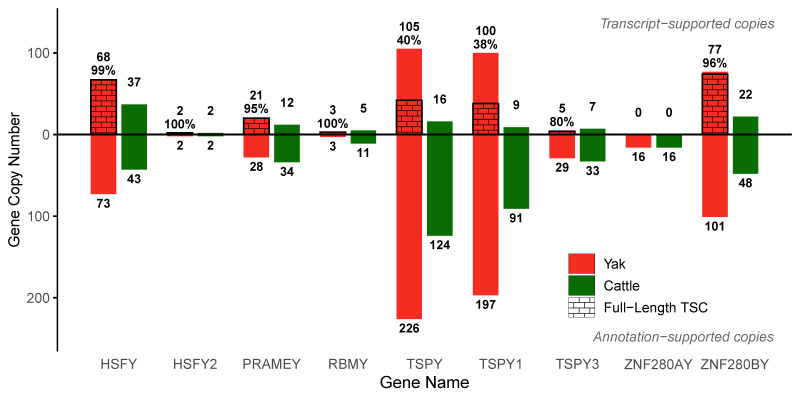
Copy number of ampliconic genes. Gene copy numbers across gene families in yak (red) and cattle (green). Upward and downward bars indicate Transcript-Supported Copies (TSCs) and Annotation-Supported Copies (ASCs), respectively. For TSCs (upward bars), total height reflects copies identified by second-generation data, while the brick-patterned sections represent high-confidence TSCs (hTSCs) further validated by third-generation data. Labels on bars denote the exact TSC counts and the percentage of hTSCs.

Drastic amplification of Y-linked ampliconic genes was observed in yaks and cattle (Figure 3, Table 1). In yak, these eight genes could be stratified into four distinct tiers based on their Annotation-Supported Copy (ASC) numbers. The most pronounced amplification was found in TSPY1 and ZNF280BY, which reached 100–200 copies. This was followed by HSFY, with an ASC range of 50–100. A third tier, comprising PRAMEY, TSPY3, and ZNF280AY, exhibited moderate copy numbers of 10–50. Finally, HSFY2 and RBMY showed the weakest amplification, with only 1–10 copies. These findings indicated that although the Y chromosome provided a favorable environment for gene amplification, the amplification process was not uniform, and the magnitude of amplification differed significantly among these genes.

However, given that a substantial portion of ASCs may represent non-functional pseudogenes that were transcriptionally silent, we proceeded to identify copies with evidence of expression. First, we defined Transcriptionally Supported Copies (TSCs) using second-generation transcriptomic data from six types of tissues (liver, lung, spleen, muscle, small intestine, and testis). Although this filtering step led to a marked reduction from the genomic ASC counts, the number of TSCs remained considerable. To obtain a more precise estimate of transcriptionally active copies, we applied a more stringent filter. We identified a subset of TSCs that were also supported by third-generation full-length transcripts, which we designated as high-confidence TSCs (hTSCs). These hTSCs were considered putative functionally active gene copies in the species. This hTSC analysis was performed using testis transcriptomic data from yaks at multiple developmental stages (6, 18, 30 months, and 4 years; Figure 1C). Since third-generation data were unavailable for cattle, our analysis for cattle was limited to the TSC level. Based on the transcriptomic profiles of yak (Figure 1C, Supplementary Figure S11) and cattle (Supplementary Figure S12), transcriptional activity of the MSY was predominantly detectable in testicular tissue. In contrast, the PAR exhibited high transcriptional activity in both the testis and other somatic tissues (liver, lung, spleen, muscle and small intestine). Furthermore, within testicular tissue, the transcriptional activity of the MSY region showed a progressive increase corresponding to sexual maturity in individuals (at 6, 18, 30 months, and 4 years; Figure 1C).

We specifically examined the TSPY gene family, which comprised TSPY1, TSPY2, TSPY3, and TSPY9. A comparison revealed that the copy number of the entire TSPY family was only marginally higher than that of the TSPY1 subfamily alone. The family as a whole exhibited an increase of only 29 ASCs, 5 TSCs, and 4 hTSCs over TSPY1. This finding strongly indicated that TSPY1 was the primary contributor to the transcriptional activity of the entire TSPY gene family on the Y chromosome.

Based on the number of hTSCs in yak relative to TSCs in cattle, we classified the eight ampliconic genes into three categories. The first category included four genes (TSPY1, ZNF280BY, HSFY, and PRAMEY), all of which exhibited a significantly higher number of active copies in yak. This dosage imbalance was most pronounced in TSPY1, where yak possessed 38 hTSCs, a 4-fold increase compared to cattle’s 9 TSCs (Table 1, Figure 3). Similarly, yak had 3.5 times more copies of ZNF280BY (74 vs. 22) and nearly double the copies for HSFY (67 vs. 37) and PRAMEY (20 vs. 12). The second category consisted of RBMY and TSPY3, where cattle had slightly higher TSC counts, possessing two more copies of RBMY and three more of TSPY3 than yak. The final group comprised HSFY2 and ZNF280AY, which were either conserved or inactive; the hTSC count for HSFY2 was low and equal in both species (two copies each), while ZNF280AY was transcriptionally inactive in both. Summarizing the findings for these eight genes, the differences were either non-existent (HSFY2, ZNF280A) or minimal (RBMY, TSPY3). The most notable observation was the drastic amplification of the four first-category genes in yak, which consequently retained far more transcriptionally active copies than cattle. For TSPY1, which exhibited both the highest hTSC count and the largest amplification fold-change relative to cattle, we further investigated its 38 hTSCs in yak. We found that 32 of these copies contained the key NAP functional domain, a number that is still 3.5 times higher than the total TSC count for TSPY1 in cattle. However, a direct comparison between hTSC and TSC presents a notable limitation. Since the TSC baseline is artificially inflated by short-read multi-mapping, the hTSC count is naturally lower. Consequently, using TSCs as a reference may lead to a conservative underestimation of the true extent of ampliconic gene expansion in yaks compared to cattle.

The TSPY1 gene family, with its extensively documented biology, serves as a compelling paradigm for dissecting the multifaceted consequences of drastic gene amplification on the Y chromosome, spanning its molecular function, complex dosage-dependent effects, evolutionary regulation, and implications for reproductive isolation. Function: TSPY1 drives spermatogonial proliferation by negatively regulating the USP7-p53 pathway. By lowering p53 levels to accelerate the G2/M transition, TSPY1 actively shifts the balance between germ cell self-renewal and apoptosis in favor of proliferation, a process essential for sustaining spermatogenesis [36]. Dosage and function: TSPY1’s contribution to spermatogenesis is governed by a non-linear dosage effect, where a moderate copy number (21–55 copies) is optimal for efficient sperm production. Deviations from this range, either through deficiency (≤20) or excess (≥56), appear to be deleterious, as both extremes confer a significantly increased susceptibility to spermatogenic impairment [76]. Dosage and expression level: The evolution of the TSPY gene family in great apes reveals a pronounced contrast: its copy number is exceptionally dynamic and diverges rapidly between species, whereas its testis expression is maintained at a high and evolutionarily constrained level, implying strong stabilizing selection on gene dosage where regulatory mechanisms actively compensate for fluctuations in copy number to maintain a stable functional output [77].

The phenomenon of male sterility in cattle–yak hybrids aligns remarkably well with these established facts. First, a key phenotype of this sterility is the depletion of spermatogonia. Given TSPY1’s critical role in promoting spermatogonial proliferation and inhibiting apoptosis, functional dysregulation of this sex-limited fertility gene provides a plausible mechanism for the depletion of the spermatogonial stem cell (SSC) pool. Second, the hybrid genome consists of equal autosomal contributions from cattle and yak, resulting in an averaging of trans-regulators from both species. Consequently, hybrids suffer from a regulatory mismatch. Hybrids with a yak sire inherit a Y chromosome with a high copy number acting as a strong cis-regulatory trait alongside intermediate trans-regulatory activity. This mirrors the deleterious effects of copy number extremes and potentially leads to overexpression relative to the hybrid background. Conversely, hybrids with a cattle sire inherit a low copy number that matches poorly with the hybrid trans-regulatory environment. This creates a risk of underexpression or silencing. Indeed, extremely low or undetectable expression of TSPY1 has been reported in cattle–yak hybrids with cattle sires [33]. We also observed that the TSC number of TSPY1 in cattle–yak decreased from nine in the sire (cattle) to three (Supplementary Figure S13). This observation confirms that the low-copy TSPY1 from cattle is unable to respond adequately to the hybrid trans-regulatory signals. Third, the evolutionary conservation of expression levels despite drastic interspecific divergence in copy number implies a tight coevolution of cis- and trans-regulators. In hybrids, this regulatory divergence generates a mismatch between the Y-linked cis-regulatory traits (copy number) and the autosomal trans-regulators, leading to systematic misregulation of these dosage-sensitive genes. Collectively, these findings provide correlative evidence linking the regulatory incompatibility arising from the drastic interspecific divergence in transcriptionally active TSPY1 copies to male sterility in cattle–yak hybrids.

Although not studied as systematically as TSPY1, the other genes (ZNF280BY, HSFY, and PRAMEY) that similarly exhibited drastic divergence in transcriptionally active copy number also demonstrated biological relevance to the phenotype of male sterility in cattle–yak hybrids. Regarding ZNF280BY, it was similarly concluded that there is no significant correlation between genomic copy number and mRNA expression levels, and its copy number (CNV) was found to be negatively correlated with testis size [78]. PRAMEY was involved in acrosome biogenesis [79] and spermatozoa maturation [80]. Finally, HSFY has been speculated to participate in spermatogenesis due to its specific expression in spermatocytes, round spermatids, and elongated spermatids, although its specific function has not yet been experimentally determined [81].

### 3.5. Conservation of Yak and Cattle Sex Chromosomes Confined to PAR

**Figure 4:**
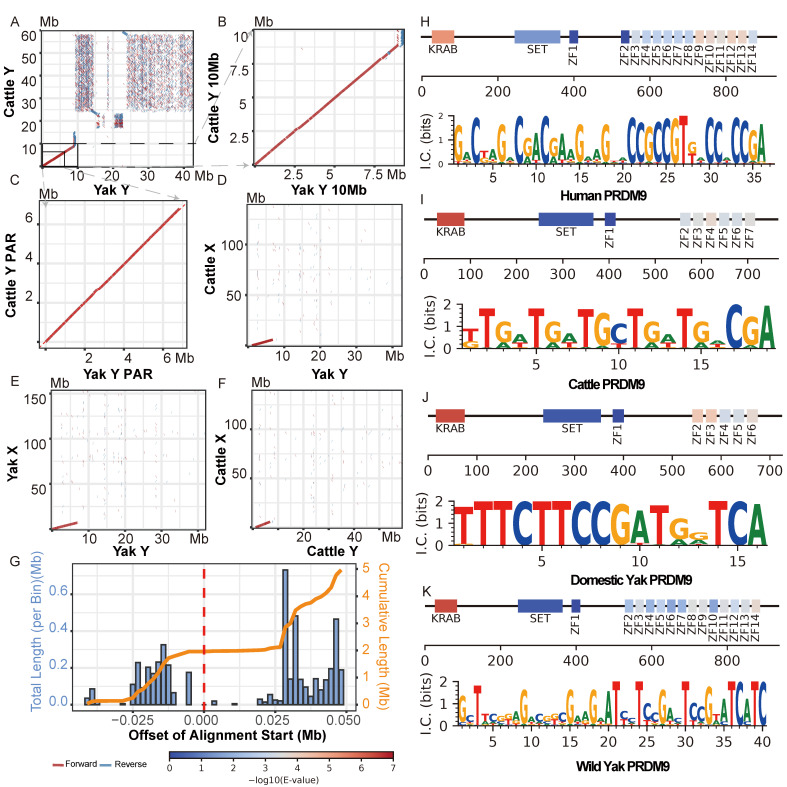
The sex chromosomal recombination region PAR and the recombination hotspot regulator PRDM9. (**A**–**G**): Dot plot and alignment shift profiling of the Yak and Cattle Y chromosome pseudoautosomal regions. (**H**–**K**): Domain diagrams and binding motif logos of human (**H**), cattle (**I**), domestic yak (**J**) and wild yak (**K**) PRDM9.

Comparative genomic alignment revealed that significant homology between the yak and cattle Y chromosomes was largely restricted to the pseudoautosomal region (PAR)—spanning approximately 6.8 Mb (Figure 4A)—with detectable similarity extending to nearly 8.75 Mb (Figure 4B). The dot plots, generated under stringent criteria (minimum alignment length 5 Kb and 99% identity), showed that the PARs of yak and cattle shared a high degree of sequence identity. However, this high identity was punctuated by numerous fine-scale gaps (Figure 4C), reflecting widespread local sequence divergence likely caused by small insertions, deletions, or accelerated regional evolution. Beyond the 8.75 Mb position, no large-scale collinear segments were observed (Figure 4A), highlighting the pronounced divergence of the Y-specific sequences between species. Furthermore, alignments between the X and Y chromosomes of yak and cattle (Figure 4D–F) confirmed that significant similarity existed exclusively within the PAR, consistent with its conserved meiotic pairing function.

The distribution of alignment shifts (reference Yak PAR start—query Cattle PAR start) exhibited a clear bimodal pattern (Figure 4G). One peak spanned from −0.025 Mb to 0 Mb, while the other, more prominent peak was concentrated between 0.025 Mb and 0.05 Mb. The presence of these distinct peaks indicated the existence of several specific, localized insertion/deletion events between the Yak and Cattle genomes. These structural variants introduced consistent offsets in the alignment coordinates, which were reflected as shifts concentrated in specific value ranges. Critically, the absolute values of these shifts (|ref_start—query_start|) were very small, with the vast majority falling below 0.05 Mb. This value (0.05 Mb) represented less than 0.7% of the total length of the PAR region (~6.8 Mb), which confirmed that the overall collinearity between the yak and cattle PAR regions remained exceptionally well preserved. This conclusion was further supported by the substantial cumulative aligned sequence length of 4,965,780 bp.

### 3.6. Distinct PRDM9 Zinc Finger Arrays and Binding Motifs in Bovines

PRDM9 is the first and most well-characterized mammalian speciation gene identified to date [82]. In mice, it is well-established that crosses between the subspecies *Mus musculus domesticus* (B6) and *Mus musculus musculus* (PWD) produce completely sterile male F1 hybrids [83]. This sterility is characterized by widespread asynapsis and meiotic arrest, a phenotype attributed to the incompatibility between their respective PRDM9 alleles [84]. Given its critical role in hybrid sterility, we performed a detailed structural analysis of the PRDM9 gene in cattle and yak. Previous studies in these species focused on the total number of zinc fingers (ZFs) without further investigating which ZFs actually determine the binding motif [71,85]. However, similar to human PRDM9 [86], we observed that the early zinc finger located away from the main array (adjacent to the SET domain) exhibited a significantly larger HMMER e-value [87] and a lower ZF score [72] compared to the main array across all bovine species (Figure 4H–K), suggesting it might not function as a typical DNA-binding C2H2 zinc finger. Consequently, by excluding this atypical domain, we determined that cattle and domestic yak contain six and five functionally active zinc fingers, respectively.

This structural divergence results in markedly distinct predicted DNA-binding motifs: the cattle motif (19 bp) is predominantly composed of thymine (T) and guanine (G), whereas the domestic yak motif (16 bp) is characterized by a prevalence of thymine (T) and cytosine (C) (Figure 4I,J). To further explore the evolutionary implications, we investigated the PRDM9 gene in wild yak (Figure 4K) and found that it possessed as many as 13 active zinc fingers—a number comparable to humans (Figure 4H) but far exceeding those in cattle and domestic yak. Although the PRDM9 binding motif of the domestic yak differs significantly from that of both wild yak and cattle, hybrids between domestic and wild yak are fully fertile, whereas those between domestic yak and cattle are sterile. These findings suggest that, unlike the mouse model, the divergence in PRDM9 binding motifs does not disrupt fertility between closely related species such as domestic and wild yaks. However, whether this divergence plays a role in the reproductive isolation of more distantly related species, such as cattle and domestic yaks, remains to be determined.

### 3.7. High Sequence Identity of Meiotic Genes Indicates a Regulatory Cause for Meiotic Arrest

Compared to yaks, cattle–yak spermatocytes display clear meiotic arrest at the pachytene stage, characterized by the absence of the XY body, persistent γH2AX and RAD51 foci on autosomes, and significantly reduced crossovers [88]. Therefore, we focused on an interspecific comparison of sequence variations in proteins regulating key events from meiotic initiation to the pachytene stage. For each specific event, we identified and examined functional modules composed of interacting proteins (Table 2). Since the strict molecular compatibility between these interacting partners is a prerequisite for their function, sequence variations may result in binding incompatibilities or reduced efficiency in the hybrid background.

We evaluated 22 key meiotic genes using annotations from Ensembl and NCBI. Among them, 19 exhibited high protein sequence identity (>95%), whereas SPO11, MDC1, and TSPYL2 initially appeared less conserved, with identities ranging from 85% to 90% in the databases. To investigate this, we aligned the cattle protein sequences of these three genes to the yak DYXZ92 assembly using miniprot. The results revealed alignment identities exceeding 99% for all three genes. Although miniprot only assesses the alignment of the cattle query to the yak genome (without accounting for potentially longer coding sequences in the native yak gene), the extremely high identity suggests that the discrepancies observed in Ensembl and NCBI are likely annotation artifacts rather than genuine divergence. We selected the DYXZ92 assembly for this analysis because it was generated using HiFi reads, offering high base-level accuracy; in our experience, other reference genomes are prone to assembly errors that lead to the false identification of critical mutations, such as frameshifts.

While sequence variation is a primary driver of incompatibility, aberrant trans-regulators can still disrupt normal meiotic progress by dysregulating target genes, even in the absence of significant sequence divergence in target genes. For instance, in our analysis, MEI1 exhibited high sequence identity between the two species (98.11% Target identity and 97.12% Query identity). However, a recent study reported that while MEI1 mRNA levels were comparable between cattle–yaks and yaks, its translation efficiency was significantly impaired in the cattle–yak’s testes [28]. This suggested that meiotic anomalies might also stem from cis-trans regulatory mismatches.

### 3.8. Curation and Benchmarking of the Yak Y Chromosome

After careful evaluation, only two assemblies—BosGru3.1 [40] and DYXZ92 [41]—were found to contain a relatively complete Y chromosome. However, the inherent complexities of sex chromosomes, particularly the vast repetitive content of the male-specific Y region (MSY) and the high sequence identity shared between the X and Y in the pseudoautosomal region (PAR), often lead to significant structural deficiencies such as major scaffolding errors and the omission of critical regions. Therefore, a systematic comparative analysis was conducted to evaluate the structural integrity of these assemblies and to validate our curated reference DYY_1.0.

**Figure 5:**
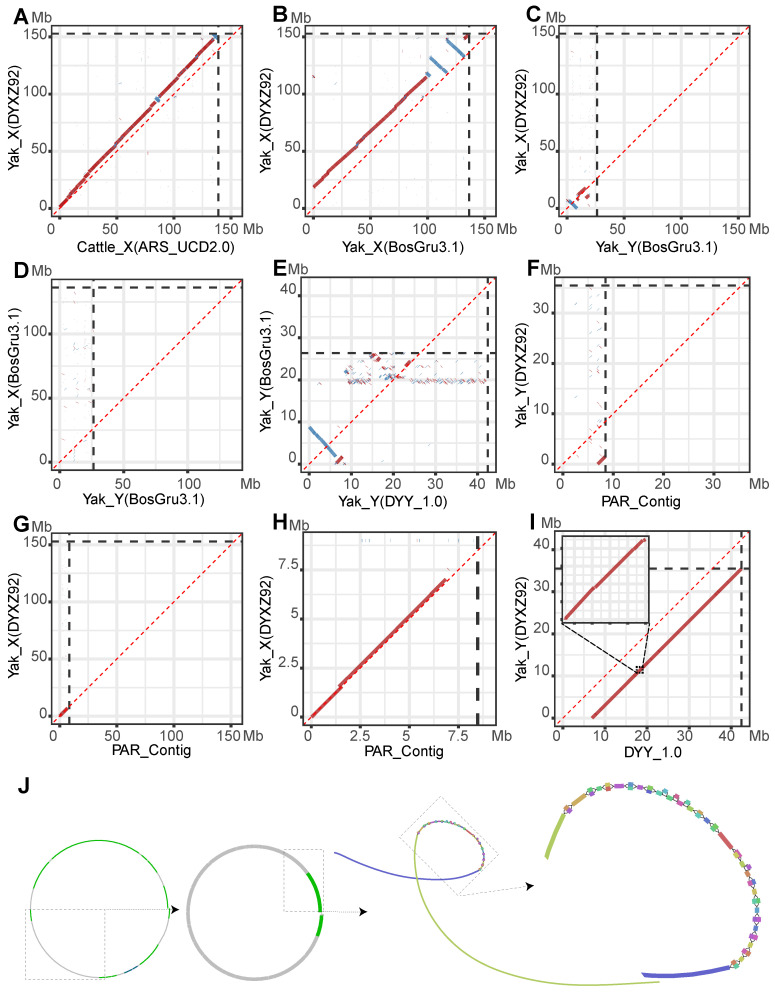
Collinearity analysis of yak and cattle sex chromosomes. (**A**): Alignment between the yak (DYXZ92) and cattle (ARS-UCD2.0) X chromosomes. (**B**–**E**): Detailed structural evaluation of the BosGru3.1 sex chromosome assembly. (**F**–**I**): Detailed structural evaluation of the DYXZ92 sex chromosome assembly. (**J**): The pan-chromosome graph of DYY_0.5.

Given the high evolutionary conservation and superior assembly quality of the X chromosome relative to the highly repetitive and rapidly evolving Y chromosome, we selected the X chromosome as a high-confidence structural baseline to accurately evaluate the Y chromosome assemblies. The DYXZ92 X chromosome (DYXZ92_X) was chosen for this role owing to its superior contiguity and completeness. To validate its large-scale structural integrity, we benchmarked it against the elite cattle reference genome, ARS-UCD2.0. The alignment displayed a high degree of collinearity between the two X chromosomes (Figure 5A), and despite a ~13.9 Mb length difference, this confirmed that DYXZ92_X could serve as a reliable reference for subsequent analyses.

With the validated DYXZ92_X as a reference, we first assessed the BosGru3.1 assembly and revealed a major structural error: a ~10 Mb segment belonging to the X chromosome had been erroneously incorporated into the Y chromosome. A comparison between the BosGru3.1 X chromosome and the DYXZ92_X (Figure 5B) revealed that BosGru3.1_X was largely collinear but exhibited a missing sequence at the PAR end. At the opposite end of the chromosome from the PAR, two large inverted segments were identified, which may indicate an improper assignment of scaffold orientation. This missing X-chromosomal segment was subsequently identified on the BosGru3.1 Y chromosome (BosGru3.1_Y), where it showed clear homology with the DYXZ92_X reference (Figure 5C). Furthermore, an alignment of BosGru3.1_Y against our final curated DYY_1.0 reference revealed a ~10 Mb central gap with no detectable similarity, directly confirming this segment as non-Y-chromosomal (Figure 5E). Therefore, given the complete lack of sequence overlap between BosGru3.1_X and BosGru3.1_Y (Figure 5D), we concluded that a segment of approximately 10 Mb, which should belong to BosGru3.1_X, had been incorporated into BosGru3.1_Y. Consequently, the BosGru3.1_Y was incorrectly structured with an inverted PAR sequence, followed by the ~10 Mb X-chromosomal segment, and a terminal ~6 Mb MSY region.

The DYXZ92 assembly exhibited greater structural integrity than BosGru3.1_Y but was still found to be incomplete. The key to its completion was a large, unplaced contig (8.48 Mb) that was identified from our Hi-C contact maps (Supplementary Figure S4), hereafter referred to as PAR_Contig. Comparative alignments against DYXZ92_X and DYXZ92_Y revealed that one terminus of PAR_Contig perfectly matched the starting region of the original DYXZ92_Y (Figure 5F), while the other terminus showed clear homology to the PAR of DYXZ92_X (Figure 5G,H). Collectively, this evidence demonstrated that PAR_Contig contained the missing PAR and its flanking sequence, acting as the critical bridge connecting the pseudoautosomal region to the male-specific region of the Y chromosome.

The construction of our final DYY_1.0 reference was a sophisticated two-stage process. In the first stage, we focused on improving the male-specific region (MSY). Using the original DYXZ92_Y assembly as a foundational backbone, we incorporated additional Y-chromosomal contigs from multiple male yak genomes via minigraph to construct a pan-chromosome graph (Figure 5J). From this graph, which captures structural variants across our cohort, we extracted a single, improved representative sequence for the MSY. As shown in Figure 5J, sequences directly derived from DYXZ92_Y (depicted in green) constituted approximately half of the constructed pan-chromosome graph. Closer inspection of sequences displaying population polymorphism (depicted in gray) revealed that they primarily consisted of short, branching haplotype segments, with no large-scale structural variations across these regions. In the second stage, we attached our previously identified PAR_Contig to the 5′ end of this improved MSY sequence and yielded the complete and curated DYY_1.0 reference assembly. To confirm that this two-stage process successfully resolved the known deficiencies without introducing structural errors, we aligned the final DYY_1.0 assembly against the original DYXZ92_Y (Figure 5I). This comparison demonstrated that the previously missing PAR region was now correctly positioned at the beginning of the DYY assembly, while the remainder of the chromosome maintained perfect collinearity with the original DYXZ92_Y sequence, barring minor InDels. This result confirmed that our curation specifically corrected the known deficiency without introducing further rearrangements, yielding a Y chromosome reference that was more complete and offered superior sequence representation compared to the previous version.

## 4. Discussion

### 4.1. A Roadmap for a Complete and Functionally Annotated Y Chromosome

Resolving the centromere and highly repetitive ampliconic regions remain the two primary bottlenecks in Y chromosome assembly. Although our work incorporated extensive data, including five published datasets and our own, the centromere and the q-arm telomere were still not resolved. Future efforts to resolve the centromere will likely require Nanopore ultra-long reads, a strategy that has proven successful in recent cattle [35], sheep [98], and goat [99] T2T assemblies. Regarding the ampliconic genes, PacBio HiFi reads successfully distinguished individual copies, but determining their functional status is challenging due to widespread pseudogenization. Identifying transcriptionally active copies requires long full-length transcriptomic reads, as short-read data cannot unambiguously assign transcripts to their gene of origin among near-identical copies.

### 4.2. Justifying the Derivation of Representative Y Chromosome via Chromosome Graph Construction

A complete assembly of the yak Y chromosome could not be achieved using the DYXZ92 reference alone, despite containing the highest-quality Y chromosome assembly available. The primary issue was that its Y chromosome assembly (DYXZ92_Y) was structurally incomplete, entirely lacking the pseudoautosomal region (PAR). Although the corresponding X chromosome contains the PAR sequence, a direct merge to complete the Y chromosome was impossible. This was due to a ~57.3 kb gap we identified between the PAR’s boundary on the X chromosome (pos. 6,858,916) and the start of the existing DYXZ92_Y sequence (pos. 6,916,176), which prevented scaffolding. This critical gap was resolved only through our de novo assembly of a new individual, Maiwa_STV1.0. This new assembly yielded a single, continuous contig (PAR_Contig) that uniquely spanned the entire missing region, containing the complete PAR and the flanking Y-specific sequence necessary to bridge the gap. By incorporating this bridging contig into the Y chromosome sequence DYY_0.5 derived from a multi-individual pangenome graph (which used DYXZ92_Y as a backbone), we generated DYY_1.0, the first structurally complete yak Y chromosome reference.

The Y chromosome is characterized by its unique patrilineal inheritance and the absence of a homologous partner [75], which precludes homologous recombination across most of its length. All these features make a single reference genome insufficient to represent the Y chromosomes’ highly divergent lineage-specific haplotypes [100]. Therefore, constructing a pan-chromosome graph to investigate population-level variations is a more suitable strategy for resolving the structure and polymorphisms of the Y chromosome [52]. To assess structural diversity across the population and generate a more representative reference, we constructed a Y-chromosome pangenome graph. Analysis of this graph revealed a critical finding: no large-scale structural variations were present among the sampled individuals in the rapidly evolving non-PAR region (Figure 5J). The graph only contained small “bubbles,” representing minor local variations, which indicated that the structural backbone of the yak Y chromosome was highly conserved. Crucially, this observed structural stability allowed us to confirm that the high gene copy numbers quantified on our representative DYY_1.0 sequence are a stable population-level characteristic, rather than an artifact of assembly or a feature specific to an individual sample. Leveraging this conserved backbone, we then generated the novel DYY_1.0 consensus sequence through a path-voting mechanism that integrated polymorphisms from all individuals. This approach ensures that the final assembly incorporates representative SNPs and small InDels, providing a more accurate and comprehensive reference for future studies on paternally inherited traits.

To evaluate the quality of our assembly, we first assessed whether the pangenome-based approach artificially inflated the chromosome length. The final DYY_1.0 assembly has a total length of 42,417,349 bp. Compared to the theoretical concatenated length of its key components (PAR_Contig and DYXZ92_Y), which is 42,404,510 bp, our assembly is slightly shorter by 12.8 kb, thus ruling out the possibility of abnormal sequence expansion. Concurrently, alignment of DYY_1.0 against the original DYXZ92_Y scaffold demonstrated a high degree of collinearity (Figure 5I), confirming that the assembly exported from the pan-chromosome graph preserved the correct chromosomal structure. Finally, to evaluate the coverage uniformity and representativeness of the DYY_1.0 sequence, we performed a coverage analysis using third-generation long reads from multiple individuals. The results showed that the reads provided uniform and near-complete coverage across the entire DYY_1.0 chromosome in samples DYXZ92 and NWIPB_DYAK_1.0, with only a few minor low-coverage regions observed in Maiwa_STV1.0 (Figure S9). This outcome provided strong evidence that DYY_1.0 was not an artificial chimera but rather a biologically meaningful reference sequence that accurately reflects the Y chromosome’s sequence features at the population level.

The minigraph algorithm [48] is inherently suited for constructing chromosome-level graphs because its implementation naturally partitions the resulting pangenome into multiple, disconnected subgraphs, each corresponding to a distinct chromosome. This structure arises necessarily from the graph chaining mechanism. Although the alignment step may identify linear chains for a single query sequence on different chromosomes, the algorithm’s subsequent step requires a pre-existing path in the graph’s topology to link them. Since the backbone establishes each chromosome as an isolated component, no such inter-chromosomal path exists. Therefore, the operational logic of the algorithm procedurally precludes the formation of edges between different chromosome-specific subgraphs, ensuring their structural independence.

### 4.3. Ensuring Comparability in Y Chromosome Analysis

To ensure the comparability of gene annotations between the yak (*Bos grunniens*) and cattle (*Bos taurus*) Y chromosomes, we implemented a harmonized and rigorous workflow at several key stages. First, using the well-annotated cattle Y chromosome as the reference, we lifted over gene annotations to the yak Y chromosome using the Liftoff tool. This process was governed by a set of stringent criteria, where a cattle gene annotation could only be successfully transferred if a match on the yak Y chromosome met all of the following conditions simultaneously: (1) it achieved a minimum sequence coverage of 50%; (2) it represented the single optimal mapping that maximized sequence identity while perfectly preserving the original gene structure (i.e., exon-intron boundaries); and (3) it was validated as a definitive one-to-one orthologous relationship through Liftoff’s conflict-resolution mechanism. This initial step ensured that homologous genes between the two species’ Y chromosomes were directly comparable in both number and identity. This rigorous process yielded a high success rate. The successful mapping of 81.84% (311/380) of cattle Y-chromosome genes to the yak Y chromosome demonstrated the high evolutionary conservation between them. Against this highly conserved background, the dramatic expansion in the copy number of key functional genes observed in yak was more likely to be a non-random evolutionary event with adaptive significance for the yak species, rather than a result of random genomic drift. Furthermore, for the de novo annotation stage, we applied BRAKER3 to both Y chromosomes. Since BRAKER3 utilized the same algorithm and criteria to identify novel genes on both chromosomes, this approach further ensured a consistent standard for the annotation of non-homologous or species-specific genes. Crucially, the high-confidence annotations generated by Liftoff were treated as the primary annotation set. Any de novo gene models from BRAKER3 that overlapped with these lifted-over annotations were subsequently discarded, ensuring that the integrity of the one-to-one orthologs was preserved. This two-tiered annotation strategy created a consistent standard for both conserved orthologs and putative species-specific genes. By establishing this robust and standardized foundation, we ensured that the subsequent comparative analysis of gene copy numbers between the two Y chromosomes would be comparable, reliable, and meaningful.

### 4.4. The Determination of Gene Copy Numbers

Prior to the availability of a complete yak Y-chromosome assembly, previous studies relied on quantitative PCR (qPCR) to estimate ampliconic gene copy numbers. However, in practice, applying qPCR to the Y chromosome is prone to significant inaccuracies. For example, as early as 2016, researchers had attempted to compare ampliconic gene copy numbers between yak and cattle in an effort to decipher the genetic basis of hybrid male sterility and observed a perplexing phenomenon that TSPY copy numbers underwent a “tremendous expansion” from sire to son [32]. Subsequently, in 2019, after optimizing primer sequences, the team corrected this artifact, demonstrating that the copy number is actually stably inherited [33]. Because qPCR quantifies only short, representative segments, its precision in complex Y-chromosomal regions is compromised by four inherent limitations: (1) Truncated copies: Short amplified segments cannot represent full-length genes, as the Y chromosome contains numerous truncated sequences retaining primer-binding sites. (2) Pseudogenization: qPCR cannot distinguish transcriptionally active genes from pseudogenes. (3) Reference bias: Relying on the cattle reference genome overlooks species-specific genetic divergence, making cattle-derived primers unreliable for yaks. (4) Sequence divergence: Mutations (e.g., SNPs) within primer-binding sites cause inefficient amplification and severe underestimation. Collectively, these limitations demonstrate that qPCR-derived copy numbers serve merely as surrogate markers for specific DNA motifs rather than accurate copy counts of target genes. These challenges profoundly underscore the imperative need for the complete yak Y-chromosome assembly presented in this study.

However, it must be noted that even with a high-quality reference genome, estimating the absolute copy number (CN) of Y-linked genes still presents inherent uncertainties. This uncertainty primarily stems from two factors. First, from a biological perspective, the Y chromosome is paternally inherited, leading to natural copy number variations among different lineages; thus, a single universal CN does not exist for a species. Second, from a technical perspective, the Y chromosome is highly enriched in repetitive sequences. Despite continuous improvements in sequencing read lengths, unresolvable long repeat arrays still trigger assembly collapse, which inevitably leads to an underestimation of CNs in the reference genome. Therefore, to achieve a relatively precise assessment of Y-linked CNs, a combined approach is essential. It requires accurately annotating the correctly assembled copies on the Y chromosome, followed by evaluating the actual CN of each individual copy using sequencing depth data. Integrating both robust genomic annotation and sequencing depth provides the most accurate copy number estimation currently possible.

### 4.5. Impaired Spermatogenesis and Testicular Development in Cattle–Yak Hybrids

Compared to cattle, yaks exhibit inherently lower spermatogenic efficiency, demonstrated not only by markedly smaller testicular volume and weight [88], but also by reduced Sertoli cell numbers and a lower germ cell proliferation rate [101]. The compromised spermatogenic capacity observed in yaks may constitute an adaptive evolutionary trade-off for survival in the extreme plateau environment characterized by hypoxia, severe cold, and forage scarcity. The cattle–yak, as hybrid offspring of cattle and yak, suffers from severe testicular atrophy and complete azoospermia [88]. Regarding testicular atrophy, it is characterized by F1 testicular weight and volume being significantly lower than those of either parental species. As for the complete azoospermia, it arises from a complete arrest of spermatogenesis caused by the cumulative effect of the cascading failure, characterized by depletion of spermatogenic substrate, blockage of differentiated spermatogonia, and pachytene arrest of spermatocytes. A simplistic hypothesis for cattle–yak sterility might attribute it solely to heterospecific sex chromosomes (an X from one species and a Y from another). If this were the case, fertility should be restored in male progeny whose sex chromosomes are homospecific. However, in current backcrossing experiments where female cattle–yak hybrids are mated with either pure cattle or yak bulls, the most favorable outcome observed in some male F1 progeny is only the presence of numerous spermatocytes and elongated spermatids [88]. Empirical observations consistently show that these backcrossed males remain sterile. This refutes the hypothesis that sterility is caused exclusively by this sex chromosome mismatch and points toward more complex genome-wide incompatibilities.

### 4.6. A Cis-Trans Regulatory Mismatch Model for Cattle–Yak Male Sterility

**Figure 6:**
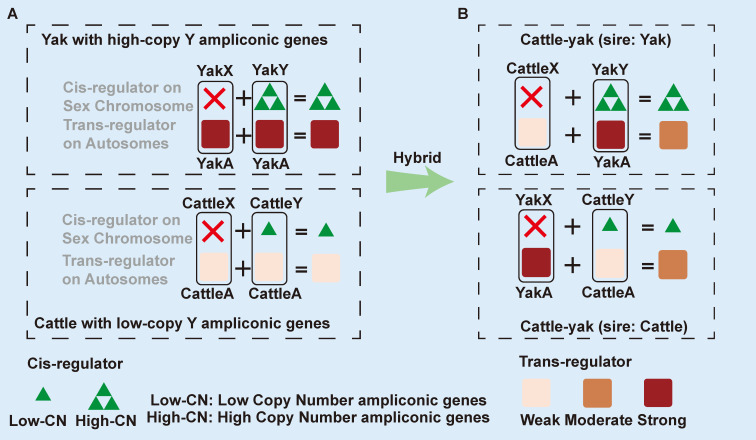
Cis-trans regulatory mismatch model elucidating male sterility in cattle–yak hybrids. (**A**): Co-evolution of cis- and trans- regulators in parental species. In yak, adaptation to the harsh plateau environment (hypoxia and cold) imposes a repressive constraint on spermatogenesis. To compensate, the yak Y chromosome underwent massive amplification of spermatogenic genes (e.g., TSPY1), evolving a High Copy Number ampliconic gene (High-CN) state. This High-CN is balanced by co-evolved Strong Trans-Regulators (Strong-TR) on autosomes to maintain homeostasis. Conversely, cattle reside in a less harsh environment and possess a Low Copy Number ampliconic gene (Low-CN) Y chromosome balanced by Weak Trans-Regulators (Weak-TR). (**B**): Hybrid breakdown. In F1 hybrids, the autosomal background is a heterozygous mix, resulting in a Moderate Trans-Regulatory (Moderate-TR) background. (**Top**) In hybrids with a yak sire, the inherited High-CN Y chromosome encounters an insufficiently repressive Moderate-TR background, leading to potential overexpression or dosage dysregulation. (**Bottom**) In hybrids with a cattle sire, the inherited Low-CN Y chromosome faces an excessively repressive Moderate-TR background, leading to transcriptional suppression or silencing. Both types of cis-trans regulatory mismatches disrupt the precise dosage required for spermatogenesis, resulting in male sterility.

By assembling the yak Y chromosome, resolving its complex ampliconic structure, and conducting comparative genomic analysis with cattle, we revealed extensive divergence in both ampliconic gene copy number and transcriptional activity of these copies between yak and cattle. Integrating these correlative insights, we proposed a “cis-trans regulatory mismatch model” as a working hypothesis to help explain the genetic mechanism of male sterility in cattle–yak hybrids (Figure 6). Inspired by the “cis-trans regulatory theory” [19], our model extended its framework to the specific context of bovine complex Y-linked ampliconic gene evolution and hybrid male sterility.

Our model is grounded in the hypothesis of compensatory co-evolution between Y-linked cis-regulators and autosomal trans-regulators (Figure 6A). In yak, adaptation to the extreme plateau environment of hypoxia and severe cold likely imposes an intrinsic physiological constraint on spermatogenesis. To counteract this suppression and maintain reproductive fitness, the yak Y chromosome appears to have undergone a compensatory evolutionary trajectory, characterized by the massive amplification of spermatogenic genes such as TSPY1 (evolving a High Copy Number ampliconic gene, or High-CN). Crucially, to prevent the deleterious effects of dosage toxicity from these amplified genes, we postulate that the yak genome has co-evolved strong repressive trans-regulators (Strong-TR) on the autosomes to maintain transcriptional homeostasis (Figure 6A, Top). In contrast, cattle, inhabiting a less extreme environment without such suppressive pressures, retain a Y chromosome with Low Copy Number ampliconic genes (Low-CN) balanced by relatively weak trans-regulators (Weak-TR) (Figure 6A, Bottom).

We propose that hybridization disrupts this precise, species-specific co-adaptation. In F1 hybrids, the autosomal background consists of a heterozygous combination of cattle and yak alleles, resulting in an intermediate or “moderate” trans-regulatory environment (Moderate-TR). This is predicted to lead to two distinct forms of dosage imbalance depending on the direction of the cross. In hybrids with a yak sire, the inherited High-CN Y chromosome encounters a Moderate-TR background that is insufficiently repressive for its high copy load, potentially leading to overexpression or dysregulation of dosage-sensitive genes (Figure 6B, Top). Conversely, in hybrids with a cattle sire, the inherited Low-CN Y chromosome is exposed to a Moderate-TR background that exerts excessive repression relative to the low copy number, creating a risk of transcriptional suppression or silencing (Figure 6B, Bottom). Indeed, in cattle–yak hybrids with cattle sires, these ampliconic genes (TSPY1, ZNF280BY, HSFY, and PRAMEY) do exhibit extremely low or undetectable expression [33].

Ultimately, we hypothesize that both scenarios may result in a failure to achieve the precise molecular dosage balance required for spermatogenesis, triggering the cascading spermatogenic failure observed in sterile hybrids. Furthermore, this model also offers a plausible explanation for the persistent sterility even when conspecific sex chromosomes are restored via backcrossing, as the underlying incompatibility presumably stems from a genome-wide mismatch between cis-regulators manifested as drastically amplified dosage-sensitive Y-linked genes and the autosomal trans-regulatory repressive background. Nevertheless, this proposed model remains a theoretical framework that requires further direct experimental validation to establish a definitive mechanistic link between Y-linked dosage-sensitive genes and cattle–yak male sterility.

## 5. Conclusions

In conclusion, we bridged a longstanding genomic gap by assembling a high-quality yak Y chromosome. By implementing a rigorous gene annotation pipeline, we established a unified standard for annotation, overcoming the technical barriers that have historically hindered accurate cross-species comparisons between yak and cattle. Our comparative analysis revealed a significant expansion of transcriptionally active spermatogenesis-related genes in yak testes compared to cattle. We hypothesize that this severe dosage imbalance leads to a “cis-trans regulatory mismatch,” which disrupts the precise molecular orchestration required for spermatogenesis and triggers the cascading spermatogenic failure observed in male cattle–yaks. Collectively, our findings challenge the traditional view of the Y chromosome as a passive element in speciation. Instead, we establish the Y chromosome as an active driver of reproductive isolation. This work not only provides a unified model as a working hypothesis to explain the mechanism of hybrid male sterility but also identifies specific molecular targets such as TSPY1 for future genetic interventions, aiming to harness the full potential of cattle–yak heterosis.

## Figures and Tables

**Table 1:** The structure and composition of yak and cattle Y chromosomes.

Features	Yak			Cattle		
	Length (bp)	Start	End	Length (bp)	Start	End
Telomere-p	18,910	1	18,910	14,685	1	14,685
PAR	6,840,006	18,911	6,858,916	6,807,695	14,686	6,822,380
Chromosome	42,417,349	1	42,417,349	59,476,289	1	59,476,289
Repeat Contents	Length (bp)	Percent		Length (bp)	Percent	
Repeat Elements	24,271,611	100%		32,203,084	100%	
Class I Retrotransposons	23,091,229	95.14		29,667,950	92.13	
LINE	18,179,882	74.90		24,287,841	75.42	
SINE	2,315,629	9.54		2,576,662	8.00	
LTR	2,595,644	10.69		2,803,373	8.71	
Penelope	74	0.00		74	0.00	
Class II DNA Transposons	1,075,376	4.43		2,402,822	7.46	
DNA	352,043	1.45		394,062	1.22	
RC-Helitron	723,333	2.98		2,008,760	6.24	
Rest	105,006	0.43		132,312	0.41	
Ampliconic Genes	ASC:TSC:hTSC			ASC:TSC	hTSC/TSC	
TSPY1	197:100:38			91:9	4.22	
ZNF280B	101:77:74			48:22	3.36	
HSFY	73:68:67			43:37	1.81	
PRAMEY	28:21:20			34:12	1.67	
RBMY	3:3:3			11:5	0.60	
TSPY3	29:5:4			33:7	0.57	
HSFY2	2:2:2			2:2	1.00	
ZNF280A	16:0:0			16:0	0.00	

**Table 2:** Orthology analysis of genes essential for meiotic recombination between cattle and yak.

No.	Description	Names	Type	Yak	Cattle	GOC Score
				Target %id	Query %id ^a^	
1	bridges histone marks on hotspots to the chromosome axis [89]	CXXC1	1-to-1	98.95	99.85	100
2	ANKRD31 interaction/ TOP6BL interaction [90]	REC114	1-to-1	99.00|98.89 ^b^		
3	PAR Targeting/Meiotic Protein Scaffolding/ REC114 interaction [90]	ANKRD31	1-to-1	98.93	96.21	100
4	MEI1 is indispensable for the recruitment of MEI4 to the meiotic chromosome axis [91]	MEI1	1-to-1	98.11	97.12	100
5	anchors the recombination machinery to the chromosome axis, preparing it for DNA cleavage [91]	MEI4	1-to-1	99.21	97.42	100
6	Promotes DSB formation via its interaction with HORMAD1 [92]	IHO1	1-to-1	98.00|100.00 ^b^		
7	DSB Formation/TOPOVIL complex [93]	SPO11	1-to-1	92.15	89.88	100
8		TOP6BL	1-to-1	97.00|100.00 ^b^		
9	Homology Search/Strand invasion [94]	RAD51	1-to-1	100.00|100.00 ^c^		
10		DMC1	1-to-1	100.00	100.00	100
11	Facilitates spreading of DDR factors for MSCI and promotes translation of meiotic genes for DSB formation. [95]	METTL16	1-to-1	100.00|99.82 ^c^		
12	Recruits DDR factors (ATR, TOPBP1, MDC1) to unsynapsed axes to initiate MSCI [95]	BRCA1	1-to-1	98.92	99.30	100
13	ATR activator and scaffold ensuring MSCI maintenance and reinforcement [96]	TOPBP1	1-to-1	100.00	98.38	100
14	Key DDR kinase initiating MSCI via H2AX phosphorylation [96]	ATR	1-to-1	99.96	99.70	100
15	DDR mediator amplifying signals to establish the sex body domain [95]	MDC1	1-to-1	100.00|87.10 ^c^		
16	Recruits USP7 to regulate epigenetic modifications on sex chromosomes [95]	SCML2	1-to-1	99.72	99.72	75
17	crossover resolution [97]	MLH1	1-to-1	100.00	100.00	100
18		MLH3	1-to-1	99.00|96.98 ^b^		
19	Prevent the premature depletion of the spermatogonial pool [36]	TSPYL5	1-to-1	100.00|100.00 ^c^		
20	Also known as TSPX, Cyclin B-CDK1 inhibitor counteracting TSPY-driven spermatogonial proliferation [37]	TSPYL2	1-to-1	93.52	88.66	50
21	The specific deubiquitination and stabilization of p53 [36]	USP7	1-to-1	97.00|100.00 ^b^		
22	The suppression of spermatogonial proliferation [36]	TP53	1-to-1	96.00|100.00 ^b^		

^a^ Query %id = Identical residues in the Alignment/Total Length of the Query Sequence. ^b^ Values represented query coverage and percent identity. These genes were incompletely annotated in Ensembl and were identified by mapping homologous protein sequences (from cattle in NCBI) to the domestic yak genome using tblastn. ^c^ Metrics corresponding to standard BLAST output (query coverage and percent identity) were calculated from the EMBOSS Needle alignment data.

## Data Availability

The High quality domestic yak Y chromosome SMU_DYY_1.0 can be accessed in the NCBI under the project PRJNA1199389.

## Supplementary Materials

The following supporting information can be downloaded at: https://www.mdpi.com/article/10.3390/biom16030471/s1, Supplementary Note S1: contig assembly using Canu; Supplementary Note S2: construction of chromosome graph; Supplementary Note S3: Identification of annotation- and tran-script-supported copies; Supplementary Note S4: Rationale for the Selection of Candidate Mei-otic Recombination Genes; Supplementary Note S5: TSC comparison across generations; Supplementary Note S6: Independent CN estimation and assembly collapse evaluation; Figure S1. Pipeline for identifying annotation-supported and transcript-supported copie; Figure S2. Collinearity Plot of Genes Transferred from Cattle to Yak Y via Liftoff; Figure S3. Identification of the pseudoautosomal, region (PAR) using Hi-C contact maps; Figure S4. Resolution of all complex branches. The red paths were the paths chosen; Figure S5. Schematic of the yak Y chromosome assembly process; Figure S6. Determination of the PAR boundary using sequence alignment and read coverage; Figure S7. Length distribution of subreads; Figure S8. Depth profiles of the de novo assembled yak Y chromosome using long reads; Figure S9. Depth profiles of the cattle (Bos taurus) Y chromosome (ARS-UCD2.0 Y) using long reads; Figure S10. Depth profiles of the centromere region of cattle (Bos taurus) Y chromosome (ARS-UCD2.0 Y) using long reads; Figure S11. Transcriptome depth profiles of the de novo assembled yak Y chromosome; Figure S12. Transcriptome depth profiles of the ARS-UCD2.0 Y chromosome; Figure S13. TSC comparison between cattle, cattle-yak and BC1; Figure S14. Read depth and CN estimation of copies of ampliconic genes; Figure S15. Chromosome-wide CN estimation of Y assemblies; Table S1. The dynamic changes in TSCs across generations.
