# Supplementary material for: Divergent Amplification of Y-Linked Dosage-Sensitive Genes Triggers Regulatory Mismatch Underlying Cattle–Yak Male Sterility
Source: Biomolecules. 2026 Mar 21;16(3):471. doi: 10.3390/biom16030471 (PMC13024182; doi:10.3390/biom16030471)
Supplement: Supplementary file 1 [file biomolecules-16-00471-s001.zip › biomolecules-4172737-supplementary.pdf]

## 1. Supplementary Notes:

### Supplementary Note S1: contig assembly using Canu

Contigs were assembled using Canu with the following parameters:
genomeSize=3g
corMinCoverage=2
minReadLength=2000
minOverLapLength=500
corOutCoverage=120
correctedErrorRate=0.035

### Supplementary Note S2: construction of chromosome graph

Commands used to construct the pan-chromosome graph were listed below
minigraph -cxggs -t 48 DYXZ92_Y.fa Maiwa_STV1.0_C.fa -o out.gfa
minigraph -cxggs -t 48 out.gfa DYPK16.genome.fa DYQH13.genome.fa DYXJ17.genome.fa DYXZ30.genome.fa -o multi.gfa

### Supplementary Note S3: Identification of annotation- and transcript-supported copies

The guiding principle of the gene annotation pipeline construction was to maximize procedural consistency, thereby ensuring the results were strictly comparable between yak and cattle. Initially, we employed Liftoff to project cattle Y annotations onto the yak Y chromosome, maximizing annotation transfer to establish a baseline for evaluating evolutionary conservation and divergence. This projection aligned our results with a widely-used standard, thus ensuring broader comparability. Consequently, the genomic regions on the yak Y chromosome annotated via this projection were treated as a final set and were excluded from any subsequent annotation or modification. Following the initial projection, our next goal was to capture potential species-specific genes arising from evolutionary divergence. To achieve this, we performed *de novo* gene prediction on the yak Y chromosome using yak-derived transcriptomic and proteomic data. However, applying this *de novo* annotation step only to the yak would create a procedural imbalance, making it difficult to distinguish genuine biological differences from artifacts of the annotation process. Crucially, by maintaining procedural identity, any potential systematic errors generated by the pipeline would be introduced indiscriminately into both genomes, preventing technical artifacts from being misidentified as true biological differences. This was especially critical because our downstream analysis focused on comparing the copy numbers of ampliconic genes between yak and cattle. Therefore, to ensure a rigorous and fair comparison, we applied the identical Braker3 pipeline to the cattle Y chromosome, using cattle-derived transcriptomic and proteomic evidence. The overall pipeline was presented in Fig 1.

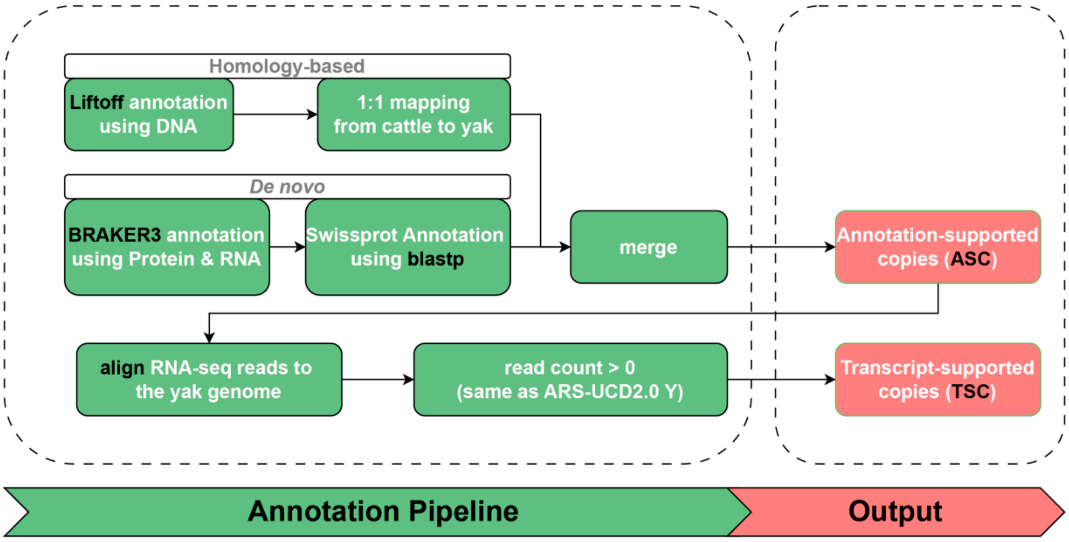


**Figure S1.** Pipeline for identifying annotation-supported and transcript-supported copies.

More specifically, Liftoff (v1.6.3) [1] was used to transfer gene annotations from the cattle Y chromosome (NC_082638.1 of ARS-UCD2.0) to our "Domestic Yak Y chromosome (DYY_1.0)" as the initial step. When selecting the gene annotation file, we identified some differences in gene counts between the authors' original study and the latest *Bos taurus* genome annotation (GFF file, GCF_002263795.3_ARS-UCD2.0_genomic.gff), which we attribute to ongoing NCBI annotation updates and therefore adopted this latest version as our reference.

Liftoff was chosen for its strict requirement for gene structure integrity and its ability to resolve paralog confusion. Gene structure integrity is critical to our study because we focus on identifying functional copies where an intact structure is a prerequisite for biological function. Another key feature of this tool is that it could map each reference gene to its single best-matched locus on the target genome. This strict one-to-one constraint enables the comparison of copy numbers between the two species. Otherwise, it would be impossible to determine whether multiple annotated loci on the target chromosome each correspond to distinct copies of an ampliconic gene from the reference, or if they were all indiscriminately identified simply due to their shared sequence similarity with a single reference copy.

In practice, we found that utilizing Liftoff's feature to annotate all potential gene copies based on a high sequence similarity criterion (-copies -sc 0.99, liftoff –sc option: minimum sequence identity in exons/CDS for which a gene is considered a copy), rather than a strict one-to-one mapping, would lead to an exceedingly high copy count. Such inflated copy numbers would severely impede subsequent analysis. For example, on the cattle Y chromosome, the lift-over annotation using the Liftoff "-copies -sc 0.99" option resulted in the identification of 362 loci whose description was "testis-specific Y-encoded protein 1-like". This number was substantially inflated compared to the 66 copies present in the official NCBI annotation. Such discrepancies would severely compromise the comparability of our results with the established reference.

Therefore, to ensure our annotation was both accurate and comparable to the NCBI standard, the Liftoff parameters were specifically defined as listed below:

$ liftoff -p 20 -f features -g only_y.gff DYY.fa ARS-UCD2.0_Y.NC_082638.1 -o dyy.gff3

notes:

1. The content of the file "features": pseudogene
2. The content of the file only_y.gff comes from the file "GCF_002263795.3_ARS-UCD2.0_genomic.gff", which is downloaded from the NCBI. The file only_y.gff only contains the chromosome "NC_082638.1" (Y chromosome).

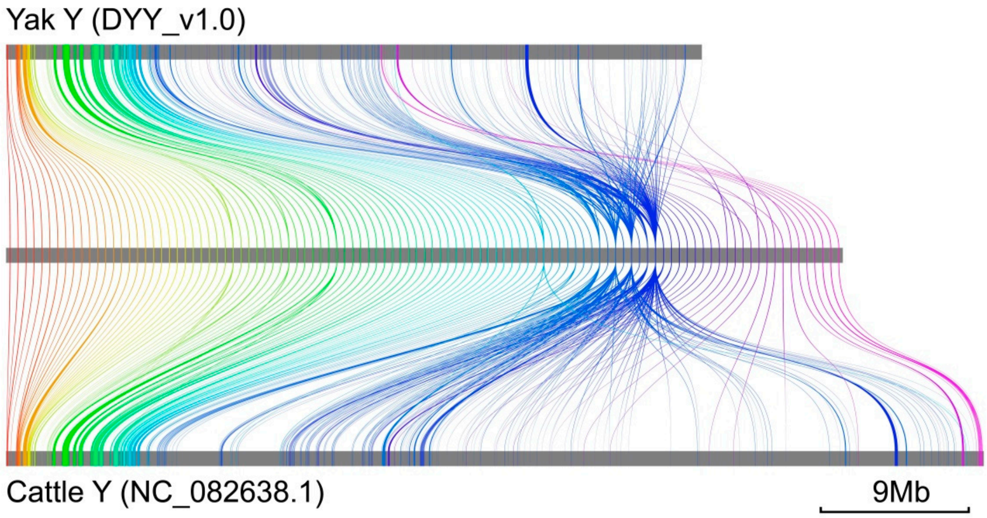


**Figure S2.** Collinearity Plot of Genes Transferred from Cattle to Yak Y via Liftoff.

After transferring, the collinearity of genes (Fig 2) was visualized with the tool linkview2 (https://yangjianshun.github.io/LINKVIEW2). Finally, out of 380 targeted records (comprising 'gene' and 'pseudogene' types), 311 were successfully lifted over from cattle to yak, representing a high transferring ratio of 81.84% (Supplementary Data S6). Crucially, only two of these mappings were flagged as low_identity (sequence identity < 50%), underscoring the high fidelity and overall quality of the successful lift-overs. As illustrated in Fig 2, genes transferred from cattle to yak maintained a high degree of collinearity within the ~10 Mb region of the PAR end. This conservation of gene order was consistent with the near-perfect sequence identity previously observed between the two species in this region.

In parallel with the Liftoff lift-over annotation, we performed *de novo* gene prediction on both the yak and cattle genomes using an identical BRAKER3 pipeline. This step was supplied with extensive extrinsic evidence (Supplementary Data S2), including protein sequences from the OrthoDB v.12 vertebrata-clade database [2], as well as from cattle (ARS-UCD2.0), mouse (GRCm39), and human (GRCh38.p14), and complemented by multi-tissue yak transcriptomic data from the liver, lung, spleen, muscle, small intestine, and testis. Since BRAKER3 primarily provides structural annotations without functional information, we subsequently annotated the predicted protein-coding genes. Specifically, the protein sequences predicted by BRAKER3 were aligned against the manually annotated and reviewed Swiss-Prot database [3] (uniprot_sprot, Release 2025_01 (05-Feb-2025)) using BLASTp with an E-value cutoff of 1e-5. The results were then filtered to retain hits with a percent identity (pident) greater than 50%. If a protein corresponding to a single

transcript had multiple annotations, the one with the highest bitscore was retained. Finally, if a single gene locus had multiple annotated transcripts (isoforms), only the transcript with the highest pident was kept for all subsequent analyses.

Finally, we merged the gene sets annotated by Liftoff and BRAKER3, respectively. For any overlapping annotations, the Liftoff-derived gene model was retained while the BRAKER3 gene model was discarded. This approach ensures consistency with the well-curated cattle reference annotation. The resulting merged gene set was then termed ASC (Annotation Supported Copies). The transcriptional activity of ASCs on the yak Y chromosome was measured by mapping transcriptomic data back to the complete yak genome assembly. To enhance the reliability of this validation, we leveraged both second-generation (short-read) and Nanopore-based full-length transcriptome data for the yak. To maintain comparability with the established cattle reference, we adopted the same threshold for transcriptional activity used in the ARS-UCD2.0 Y chromosome study [4]. Accordingly, a gene was considered transcriptionally active if any expression was detected (transcript count > 0).

In the annotation file, most records contain only functional descriptions and have unofficial or placeholder gene names. To ensure consistency with the cattle study, we matched the coordinates associated with their provided gene names to the coordinates of our functional descriptions, thereby assigning these descriptions a unified gene name. The NCBI annotation results encompassed both genes and pseudogenes. The 'gene' category is further subdivided based on the presence or absence of a '-like' suffix in the gene description, distinguishing canonical genes from their putative homologs. In cattle (ARS-UCD2.0) Y's research [5], when counting gene copy numbers, descriptions for both "testis-specific Y-encoded protein 1-like" and "testis-specific Y-encoded protein 1" were classified under the protein-coding gene TSPY1. For comparability, we have adopted the same counting method. Consequently, the mapping relationship between the descriptions and gene names used in our quantification was shown in the Supplementary Data S7 below.

## Supplementary Note S4: Rationale for the Selection of Candidate Meiotic Recombination Genes

In sterile male cattle-yak hybrids (*Bos taurus* × *Bos grunniens*), spermatogenesis is consistently arrested at the pachytene stage of meiosis prophase I [6,7]. While the formation of meiotic double-strand breaks (DSBs) in male cattle-yak appears unimpaired, the subsequent repair process is abnormal. This is evidenced by cytological analysis of pachytene spermatocytes, where markers for DNA damage ($\gamma$H2AX) and repair intermediates (RAD51) persistently localize to the autosomes. This contrasts sharply with the pattern in fertile parental yaks, where these signals are efficiently cleared from autosomes and become predominantly confined to the sex chromosomes [8]. A remarkably similar cellular pathology underlies hybrid male sterility in the well-characterized cross between *Mus musculus musculus* (PWD) and *M. m. domesticus* (B6) mouse strains. In this model, the pachytene arrest is triggered by a genetic incompatibility between parental alleles of the meiotic regulator PRDM9 and the X-linked locus Hstx2, which results in the complete absence of two critical COMPASS complex proteins, EWSR1 and CXXC1, and ultimately leads to azoospermia and testicular atrophy [9].

The defects observed in both cattle-yak and mouse hybrids serve as compelling examples of the Dobzhansky-Muller model of incompatibility [10]. This evolutionary framework suggests that alleles diverging in isolated populations, while benign in their native genetic contexts, can engage in deleterious epistatic interactions when combined in a hybrid genome, thereby disrupting essential biological pathways like meiosis. Given that such incompatibilities are rooted in genetic divergence, we sought to investigate the potential molecular basis for these meiotic failures by conducting a detailed comparative analysis of the protein sequences encoded by key functional genes involved in the meiotic recombination of the meiotic prophase I.

Meiotic recombination, the process that ensures genetic exchange and proper chromosome disjunction, is executed by a series of protein complexes responsible for key sequential steps, including DSB formation by SPO11, strand invasion by RAD51/DMC1, and crossover resolution primarily by the MLH1-MLH3 nuclease complex [11]. Focusing on the initial step, the induction of DSBs is primarily catalyzed by the evolutionarily conserved topoisomerase-like protein SPO11, acting in synergy with TopoVIBL. However, a critical prerequisite for their formation is the precise spatial determination of where these breaks should occur. In most mammals, this task of hotspot specification on autosomes is predominantly orchestrated by PRDM9. As a sequence-specific histone methyltransferase, PRDM9 binds to distinct DNA motifs and licenses these sites by depositing epigenetic marks on local chromatin [12]. The effective recruitment and activation of the SPO11-TopoVIBL machinery at these licensed sites is, in turn, orchestrated by a crucial multi-protein complex [13]. Central to this multi-protein complex is the vertebrate-specific protein ANKRD31, which functions as a modular scaffold. ANKRD31 interacts with several other essential pro-DSB factors, including REC114, MEI1, MEI4, and IHO1, collectively forming what has been termed the RMMAI (REC114-MEI1-MEI4-ANKRD31-IHO1) complex [14]. The function of this entire assembly is particularly critical during male meiosis, where ANKRD31 is responsible for targeting DSBs to the pseudoautosomal region (PAR) of the X and Y chromosomes, a process essential for their pairing and subsequent segregation [12].

A pivotal early event in meiotic recombination is the stable anchoring of pre-DSB RMMAI proteins (REC114, MEI1, MEI4, ANKRD31 and IHO1) to the chromosome axis. On autosomes, this process is critically dependent on HORMAD1-IHO1 interaction [15]. In contrast, within the PAR of sex chromosomes in the mouse, this anchoring occurs independently of HORMAD1 and is instead mediated by mo-2 minisatellite arrays [14].

Once properly positioned, these pre-DSB RMMAI proteins are thought to recruit or activate the SPO11-TOPOVIBL complex, which is responsible for catalyzing the formation of DNA double-strand breaks (DSBs) [16].

The DSB hotspots in the autosomes is mainly regulated by PRDM9 [17]. And the formation of DSBs within the PAR doesn't entirely depend on the PRDM9 [14]. When SPO11β was exclusively expressed, global DSB levels exhibited no marked alterations, but DSB formation within the PAR was notably diminished. In contrast, sole expression of SPO11$\alpha$ resulted in minimal DSB formation; however, the co-expression of SPO11$\alpha$ with SPO11β significantly enhanced DSB formation frequency with the PAR [18]. The SPO11β isoform alone suffices to orchestrate the majority of DSB formation, facilitate autosomal recombination, and sustain female fertility. Nevertheless, efficient X-Y chromosomal pairing and male fertility critically depend on the cooperative function of SPO11$\alpha$ and SPO11β [19].

The pairing of autosomes is generally less challenging compared to sex chromosomes, as pairing of the X and Y chromosomes occurs only at the PAR and the shorter PAR limits opportunities for homologous recombination [20]. During meiosis, sex chromosomes exhibit unique pairing dynamics, relying exclusively on PAR-mediated homologous interactions to achieve accurate recombination and synapsis [21]. Unsynapsed regions of sex chromosomes harbor "pachytene-lethal" genes, such as ZFY, whose expression disrupts meiotic progression by inducing arrest at the pachytene stage. To circumvent this, meiotic sex chromosome inactivation (MSCI) is activated, primarily through the formation of the transcriptionally silenced XY body, which ensures the suppression of deleterious gene expression in unsynapsed chromatin [18] .

## Supplementary Note S5: TSC comparison across generations

We completely agree that hybrid expression data would substantially strengthen the proposed "cis-trans regulatory mismatch" interpretation. Fortunately, RNA-seq data of the testes of F1 cattle-yak hybrids (sire: cattle, n=3, SRR20334286, SRR20334287, SRR20334288) and the BC1 backcross generation (sire: cattle, n=3, SAMC3870920, SAMC3870921, SAMC3870922) is publicly available, which allowed us to analyze the number of Transcript-Supported Copies (TSCs) across these generations. As shown in the Figure S13, the dynamic changes in TSCs across the parental cattle, F1 hybrids, and BC1 generation can be broadly categorized into four distinct modes (Table S1).

**Table S1.** The dynamic changes in TSCs across generations.

| | Cattle-yak | BC1 | Genes |
|---|---|---|---|
| Mode 1 | Down | Up | HSFY2, TSPY<br>TSPY1, HSFY |
| Mode 2 | Up | Down | RBMY |
| Mode 3 | Unchanged | Up/Down | TSPY3， PRAMEY |
| Mode 4 | Unchanged | Unchanged | ZNF280BY<br>ZNF280AY |

We specifically focused on the four ampliconic genes (TSPY1, HSFY, PRAMEY, and ZNF280BY) that exhibit massive amplification in yaks. Notably, TSPY1 and HSFY strictly follow Mode 1 (initial downregulation in F1, followed by upregulation/recovery in BC1). Meanwhile, PRAMEY and ZNF280BY largely maintain unchanged TSC levels in the hybrids (Modes 3 and 4). The mode 1 pattern observed for TSPY1 and HSFY is highly consistent with, and provides robust evidence for, our "cis-trans regulatory mismatch" hypothesis.

## Supplementary Note S6: Independent CN estimation and assembly collapse evaluation

Briefly, we retrieved long-read sequencing data from two male cattle (Braunvieh and RGVxSIM) and two male yaks (NWIPB_DYAK_1.0 and DYXZ92). Reads were mapped to their respective genome references (including the Y chromosome) using minimap2. The baseline diploid depth was estimated using autosomal chromosome 1. Assuming the haploid Y chromosome should exhibit half the autosomal depth, the locus-specific CN was estimated using the following formula: CN = round (read_depth / average_depth of the Y chromosome)

The visualizations of the read depth and estimated CNs for these genes are presented in Figure S14A-D (dots represent read depth; bars represent the estimated CN). In cattle (Figure S14A, S14B): We observed that the average depth for most copies is approximately 1X, with small part of loci exhibiting a CN > 2. Additionally, several copies present in the Y reference assembly were absent in the mapped individuals, indicating lineage-specific loss within cattle. In yaks (Figure S14C, S14D): In stark contrast, almost all copies could be detected in the yak Y reference assembly. Furthermore, a significant proportion of these ampliconic gene copies exhibited an estimated CN > 2.

Furthermore, we examined the chromosome-wide read depth and estimated the copy number (CN) across the entire Y chromosome (Figure S15A, S15B). In both cattle and yaks, approximately 25%-35% of the Y chromosome regions exhibited an estimated CN>1, indicating the potential presence of assembly collapse. Although the total proportion of these collapsed regions is comparable between the two species' Y chromosome assemblies, their spatial distribution differs: in cattle, the collapsed regions are predominantly concentrated in the central segment of the chromosome, whereas in yaks, they are more dispersedly distributed.

Since the estimated CNs for the ampliconic gene copies in yaks remain significantly higher than those in cattle, the presence of these collapsed regions does not alter our primary findings: ampliconic genes have undergone a much more drastic amplification in yaks than cattle.

## 2. Supplementary Figures

**(A) Large-scale (~850 Mb window)**

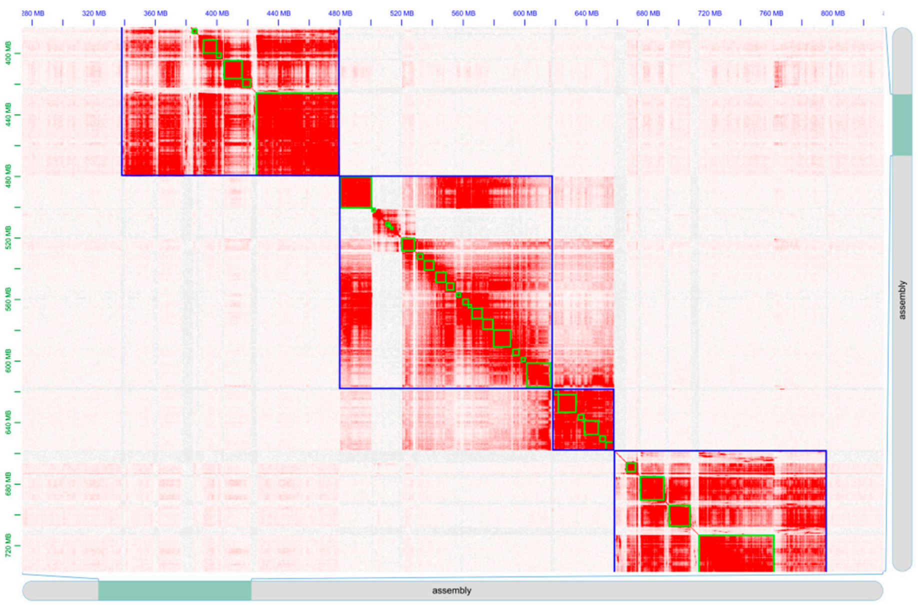


**(B) Local-scale (55 Mb window)**

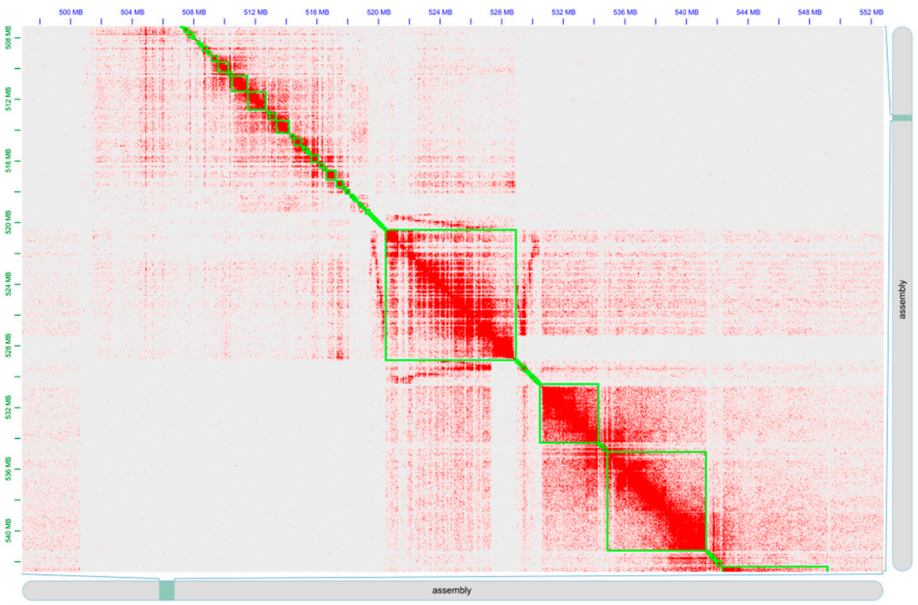


**Figure S3. Identification of the pseudoautosomal region (PAR) using Hi-C contact maps.**

**(A)**

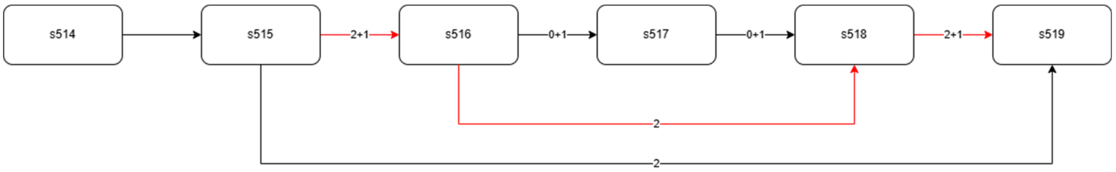


**(B)**

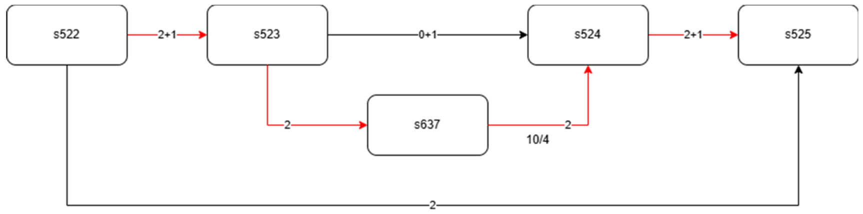


**(C)**

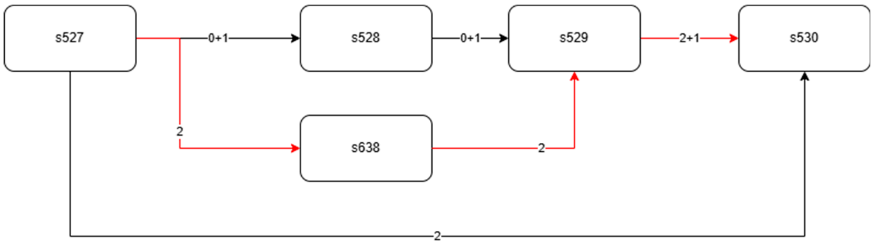


**(D)**

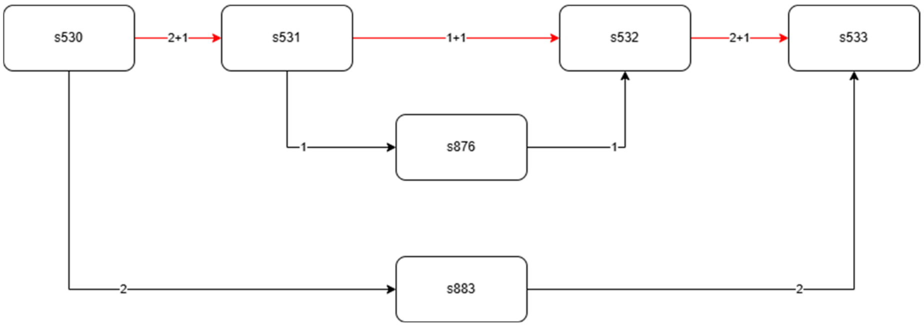


**(E)**

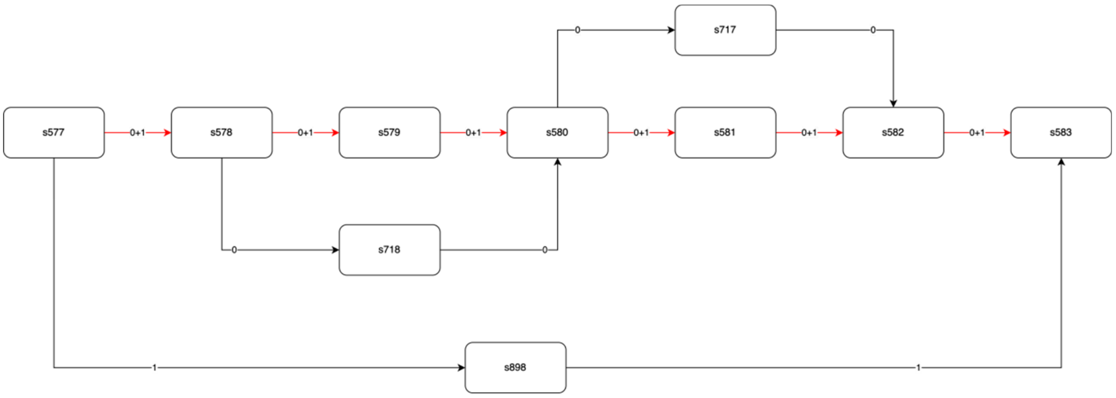


**Figure S4. Resolution of all complex branches.** The red paths were the paths chosen.

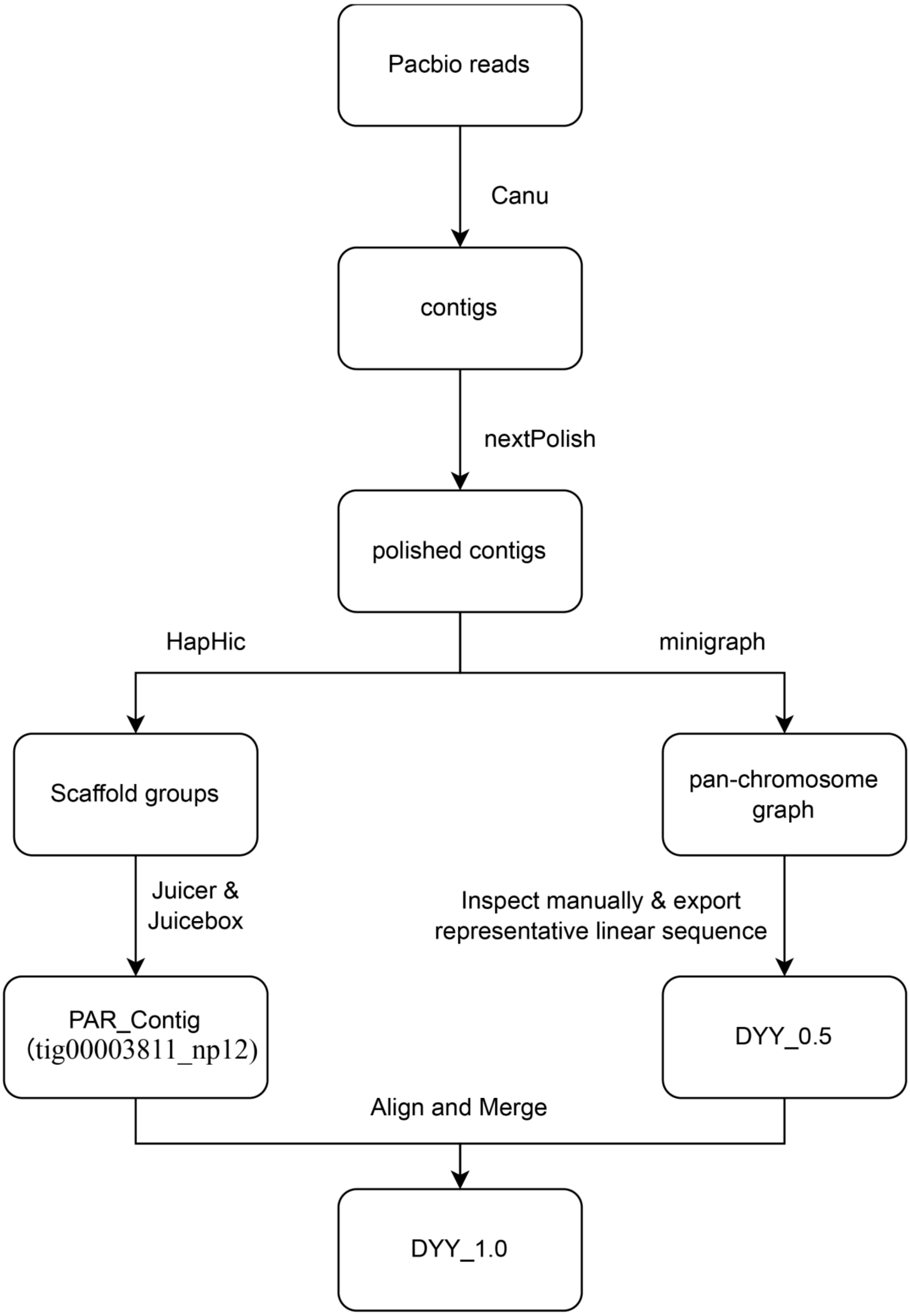


**Figure S5. Schematic of the yak Y chromosome assembly process.**

(A)

```
DYXZ92_X        1351 GGAGTGTGGTGGTCTGTGCACGGGCTGGAAAAGTTTTCAGAGATGAGGCA   1400
                     ||||||||||||||.||||||.||.|||||||||||.|||||||||||||
PAR_Contig      3540 GGAGTGTGGTGGTCCGTGCACCGGTTGGAAAAGTTTCCAGAGATGAGGCA   3589

DYXZ92_X        1401 GAATGAGAGGAGAGGAGAGTTTATCAGAG-------AGGGAGACGCCTTT   1443
                     |||||||||||||.||||||.||||.|||  Gap1 .||||.|.||..||
PAR_Contig      3590 GAATGAGAGGAGAAGAGAGTGTATCGGAGTGCAGTATGGGATATGCTGTT   3639

DYXZ92_X        1444 AGAACAGGGGGCCGGTCAA-GGAGAGCCAGCCCCTTTGATTGGCTAGCAG   1492
                     ||||||||||||||||||| |||||||||.||||||||||.|||||||||
PAR_Contig      3640 AGAACAGGGGGCCGGTCAAGGGAGAGCCAACCCCTTTGATGGGCTAGCAG   3689

DYXZ92_X        1493 CCCATTTTTATAGCCTTAAGACAGAAAATTCCTACTAGAAGGGTGGCATT   1542
                     |||||||||||||.||.|||||||||||||||||||||||||.|||||||
PAR_Contig      3690 CCCATTTTTATAGTCTCAAGACAGAAAATTCCTACTAGAAGGATGGCATT   3739

DYXZ92_X        1543 AGGTGATTGGTTAGGATGCTGTGTGTGATAAGTTGCTTCACTCGTGTCCG   1592
                     ||||||||.|||||||||||.|||||||||||||||||||..|.||||.|
PAR_Contig      3740 AGGTGATTAGTTAGGATGCTATGTGTGATAAGTTGCTTCAGCCATGTCTG   3789

DYXZ92_X        1593 ACTCTTTCTGTGATCCTATGGGCTGTAGCCCACCAGGCTCCTGTGTCCCT   1642
                     |||.|||.||..|||||||||||||||..||| ||||.||||.|||||..
PAR_Contig      3790 ACTGTTTTTGCCATCCTATGGGCTGTACTCCA-CAGGTTCCTCTGTCCAC   3838

DYXZ92_X        1643 GGGATTCTCCAGGCAAGAATACTGGAGTGGGTTGCCATGCCCTCCTCCAG   1692
                     ||||||||||||||||.||||||||||||||||.|||||||.||||||||
PAR_Contig      3839 GGGATTCTCCAGGCAAAAATACTGGAGTGGGTTCCCATGCCTTCCTCCAG   3888

DYXZ92_X        1693 GGGATCTTCCCAACCTAGGGATCAAACCCGAGTCTTATGTCTCATGCA   1742
                     |||||||||||||...|||||||.|||.|  Gap2   |.|||||...||
PAR_Contig      3889 GGGATCTTCCCAAATCAGGGATCGAACAC---------AGGTCTCCCCA   3929
                                              3917

DYXZ92_X        1743 TTGGCAAGCAG-TTTGTTTAC---CTCAAGTGCCACTTGAGAACCCCCTA   1788
                     .|     |||| .||.|||||   ||   |.|||||..|.|||.||||.|
PAR_Contig      3930 CT-----GCAGAATTCTTTACTAGCT---GAGCCACAAGGGAAGCCCCGA   3971
```

(B)

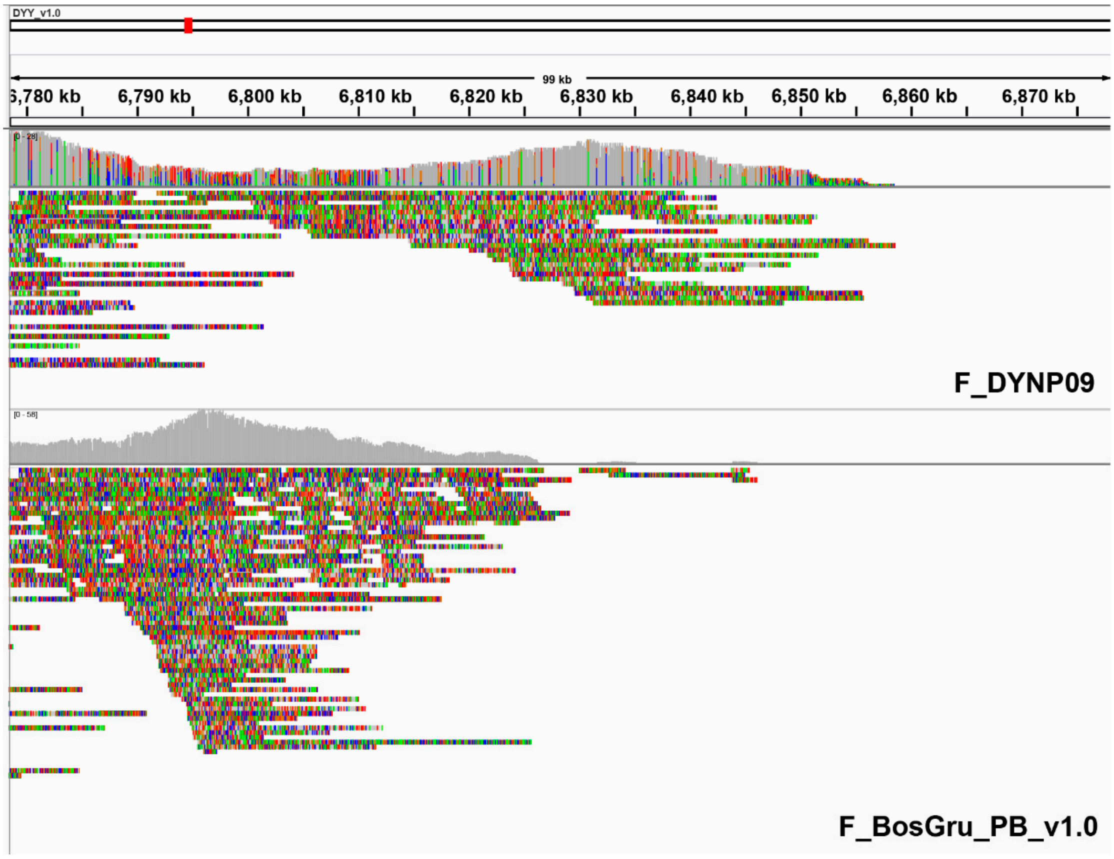


(C)

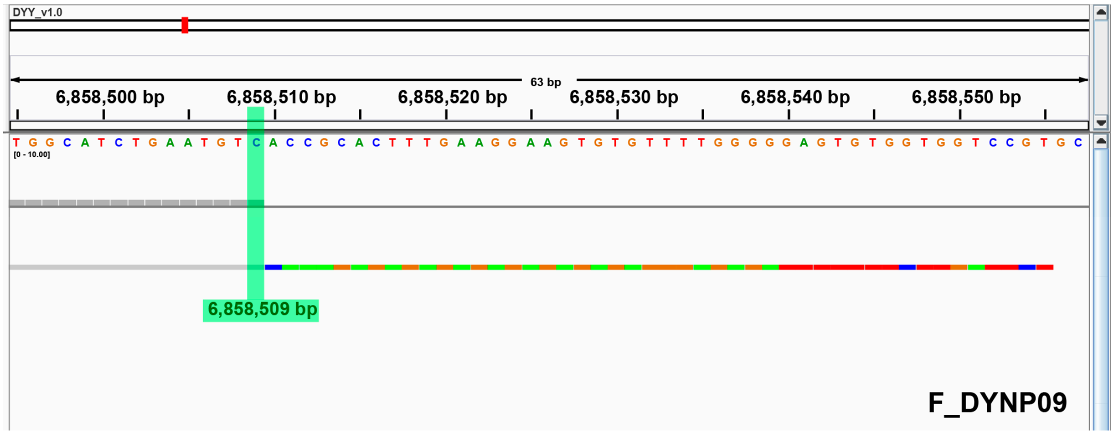


**Figure S6. Determination of the PAR boundary using sequence alignment and read coverage.**

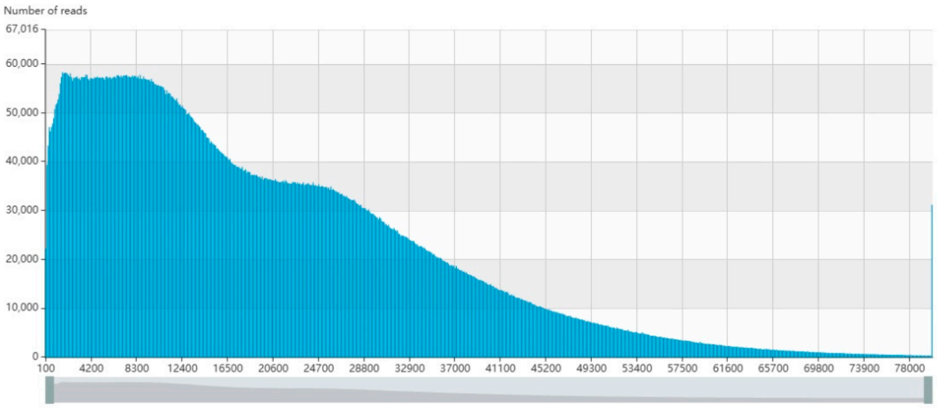


**Figure S7. Length distribution of subreads.**

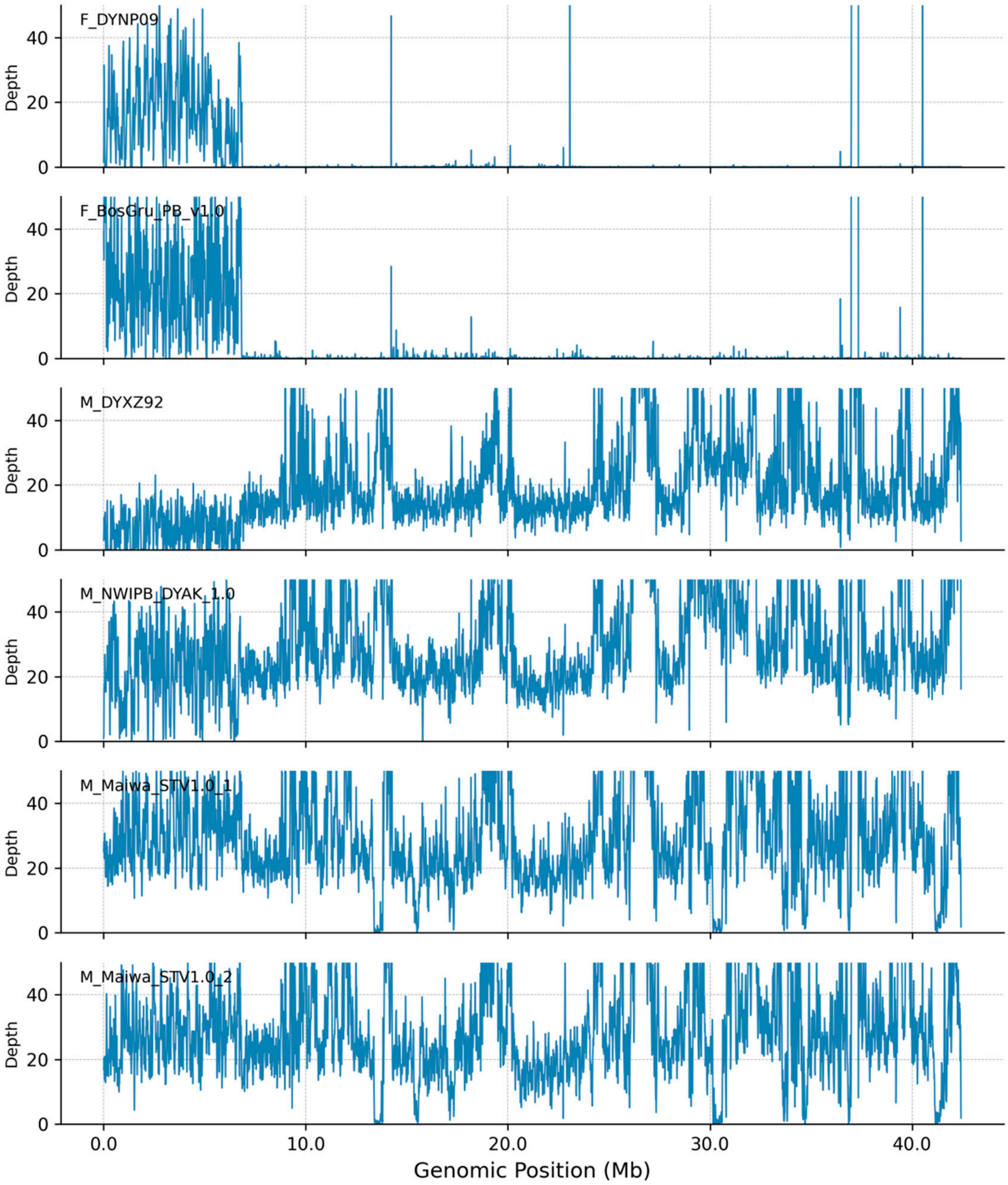


**Figure S8. Depth profiles of the *de novo* assembled yak Y chromosome using long reads.**

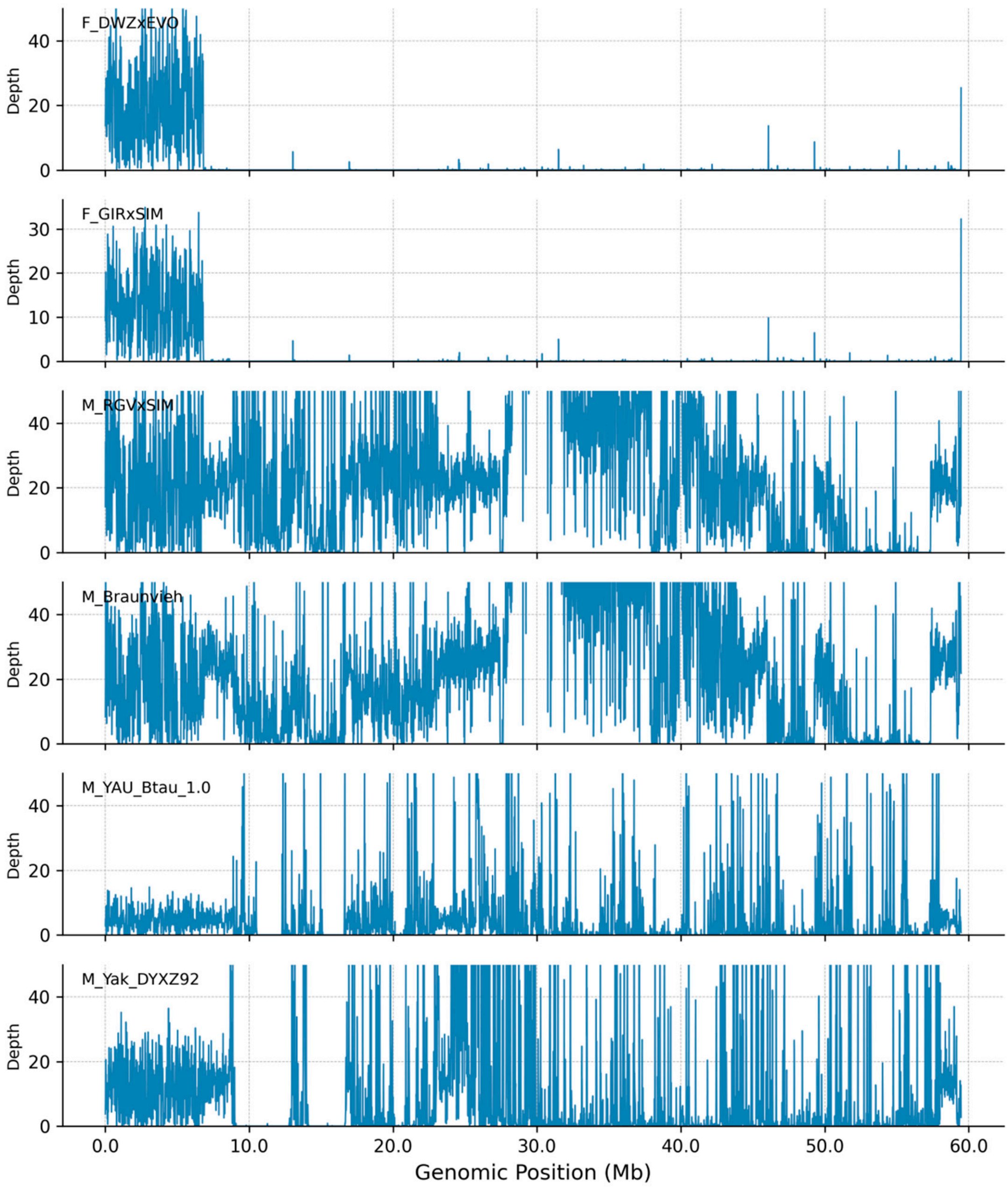


**Figure S9. Depth profiles of the cattle (*Bos taurus*) Y chromosome (ARS-UCD2.0 Y) using long reads.**

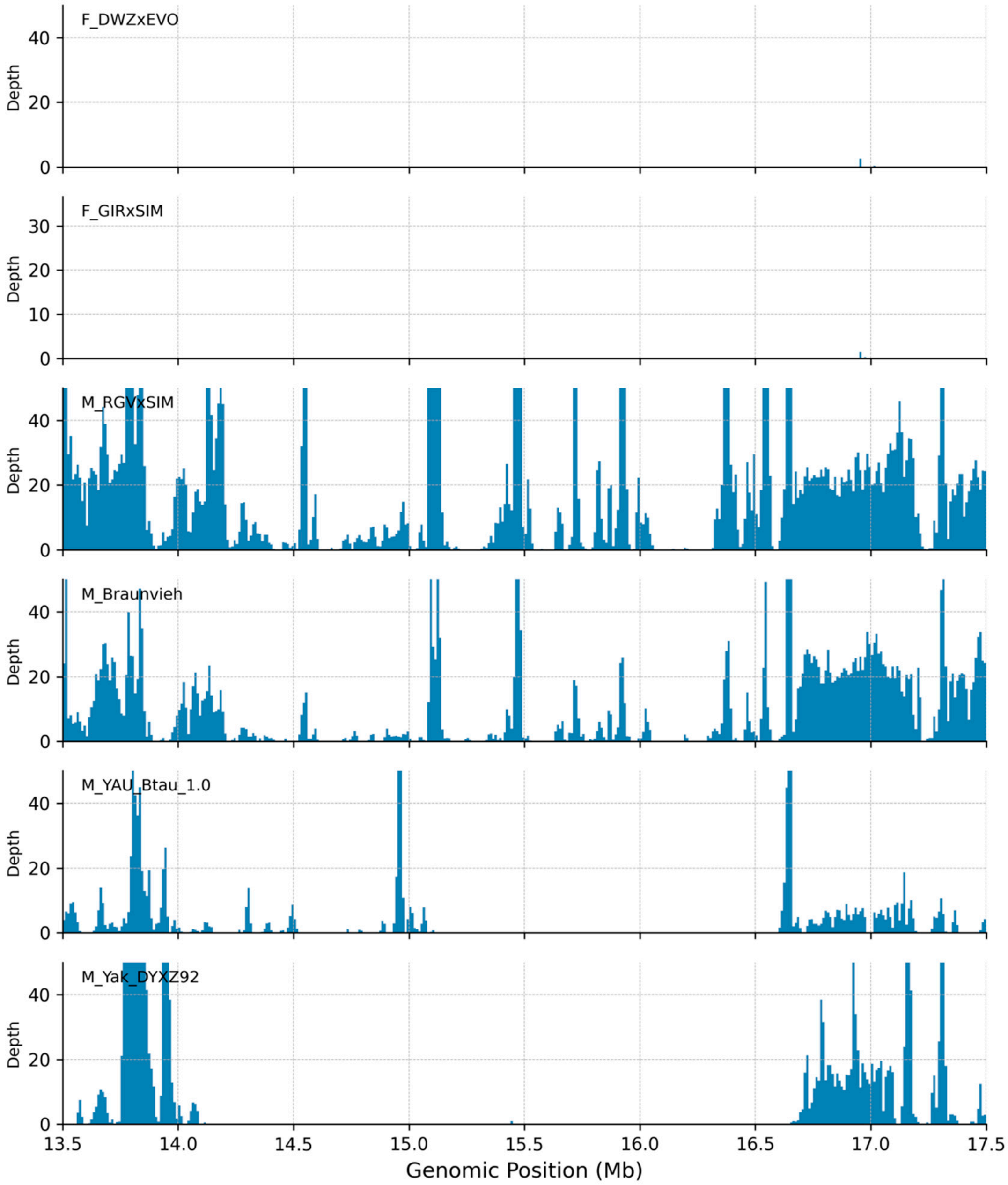


**Figure S10. Depth profiles of the centromere region of cattle (*Bos taurus*) Y chromosome (ARS-UCD2.0 Y) using long reads.**

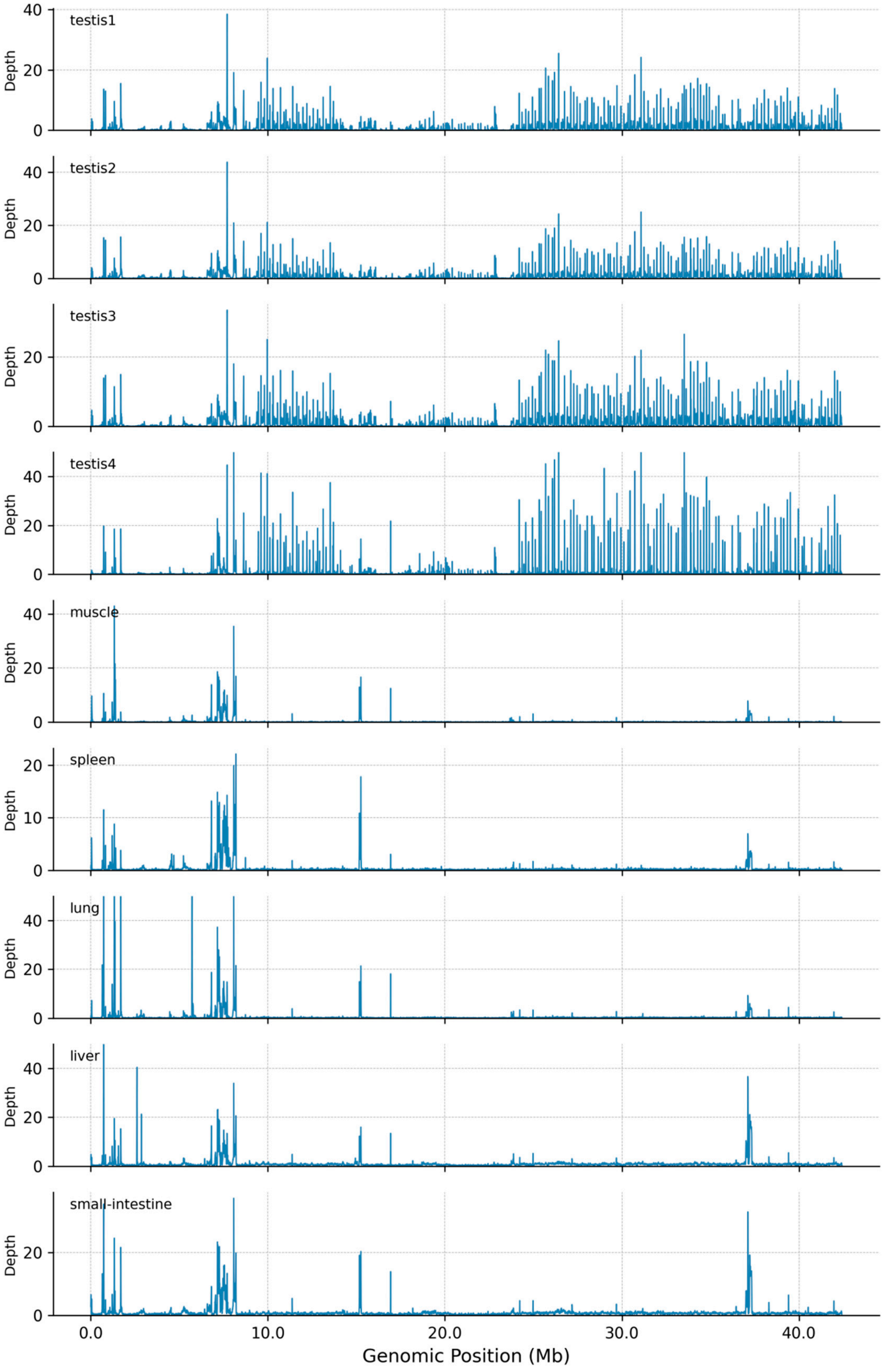


**Figure S11. Transcriptome depth profiles of the *de novo* assembled yak Y chromosome.**

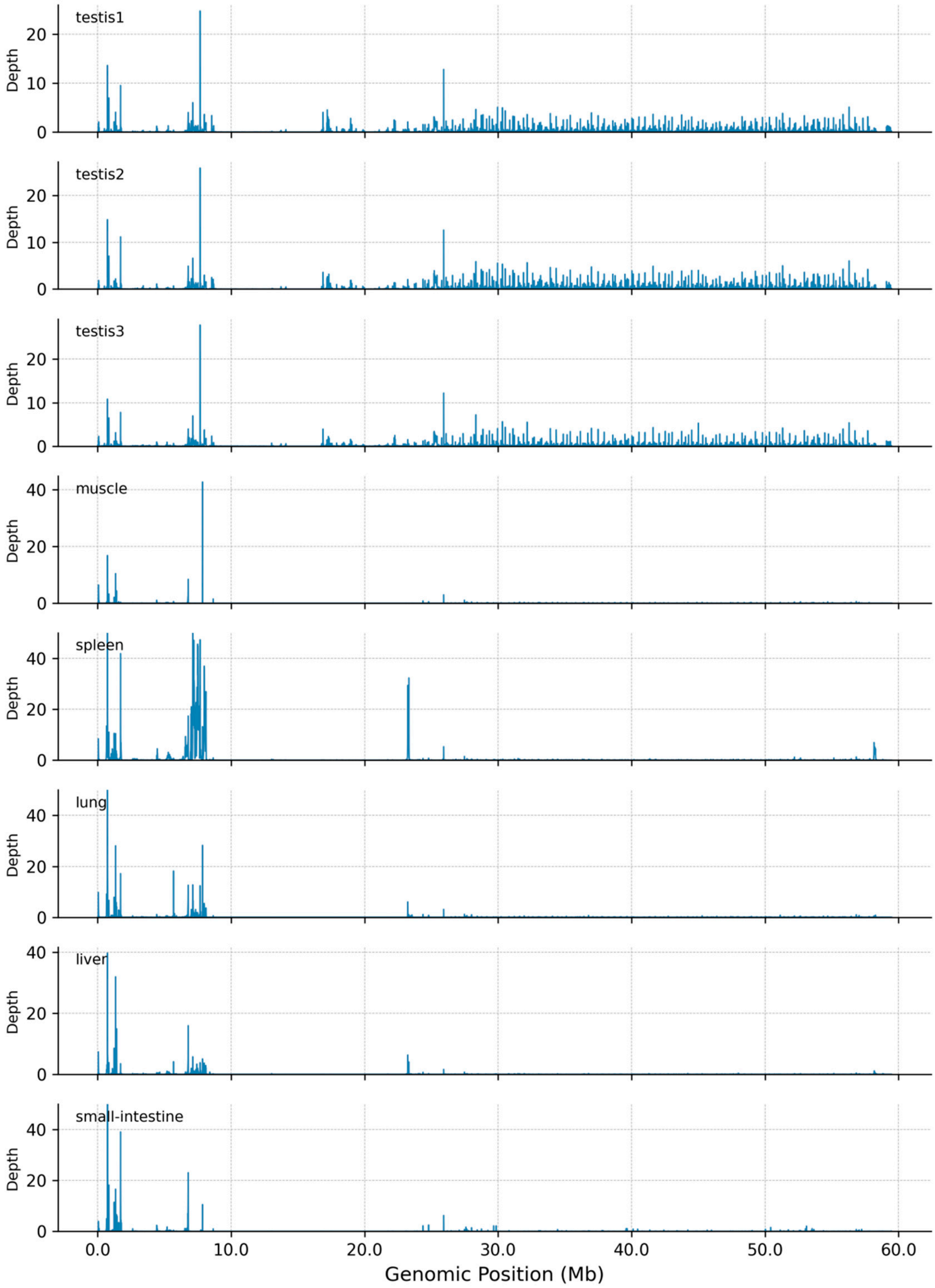


**Figure S12. Transcriptome depth profiles of the ARS-UCD2.0 Y chromosome.**

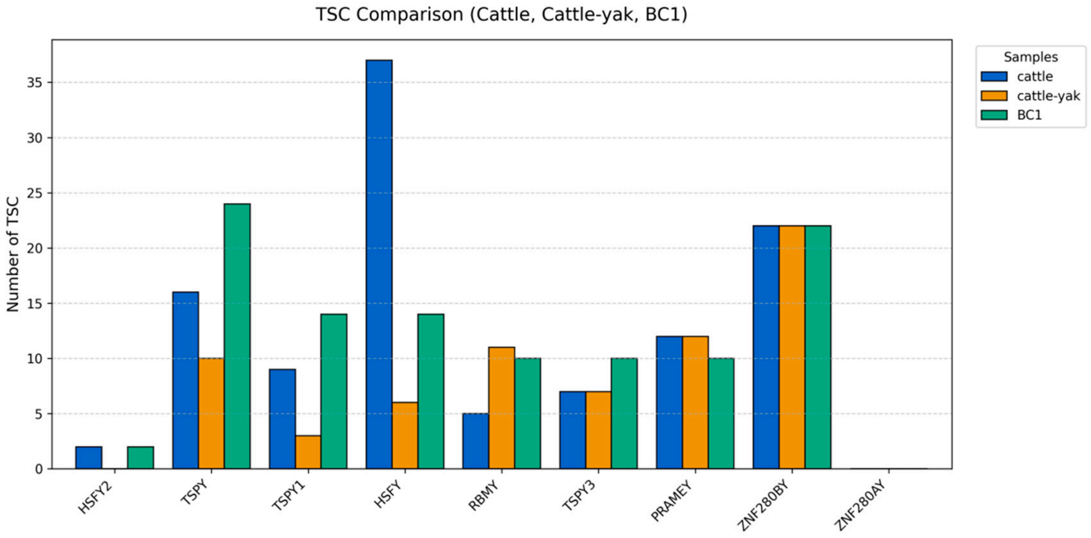


**Figure S13. TSC comparison between cattle, cattle-yak and BC1.**

(A)

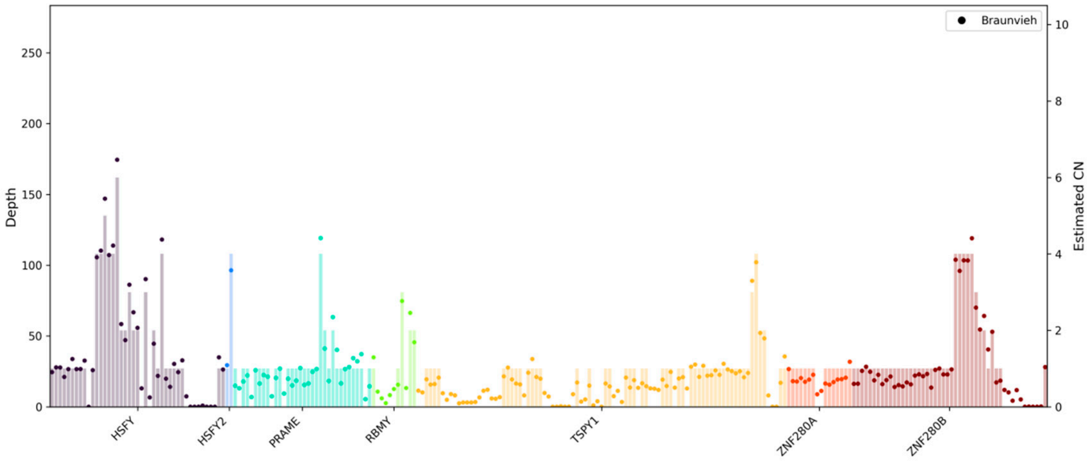


(B)

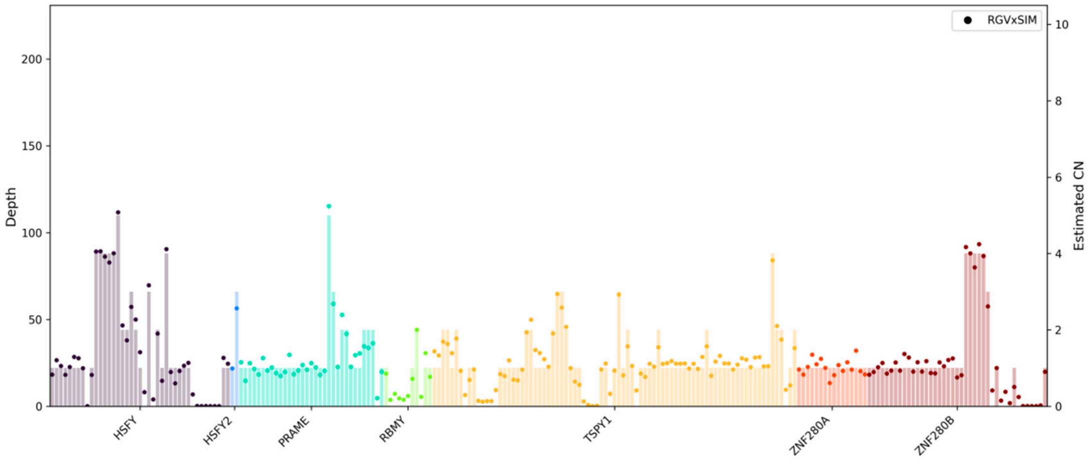


(C)

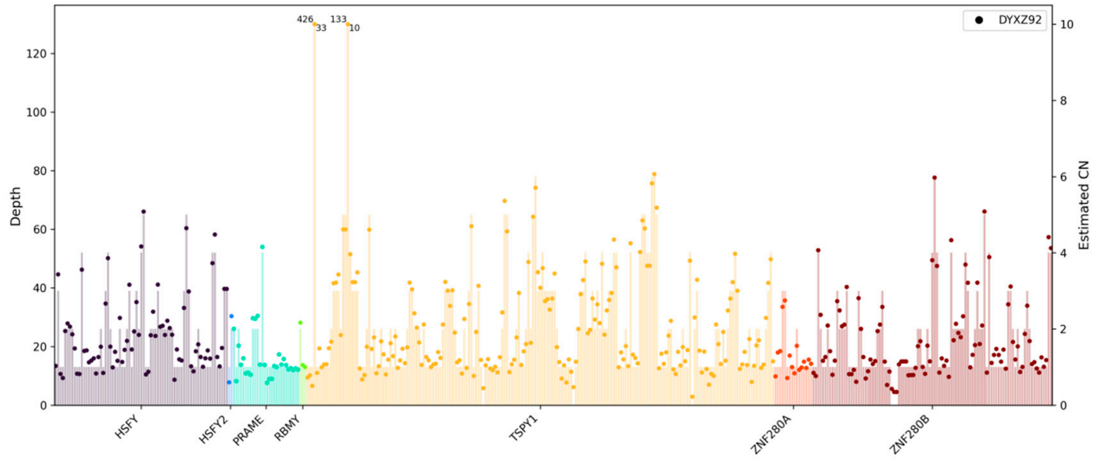


(D)

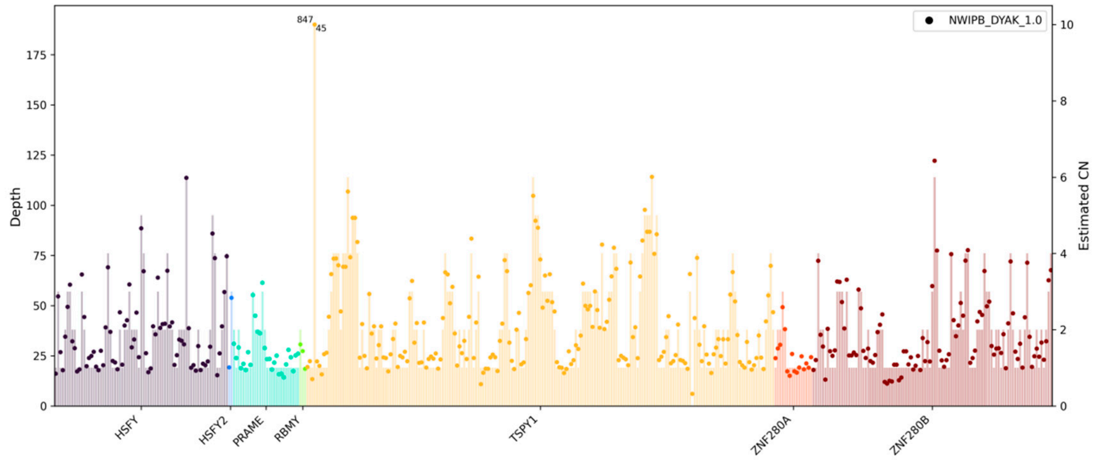


**Figure S14. Read depth and CN estimation of copies of ampliconic genes.**

(A) Yak (DYXZ92)

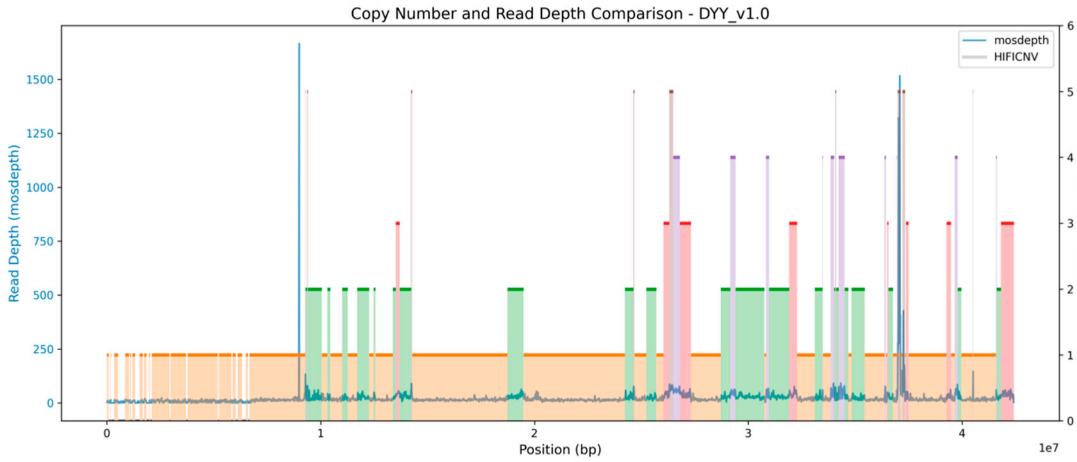


(B) Cattle (RGVxSIM)

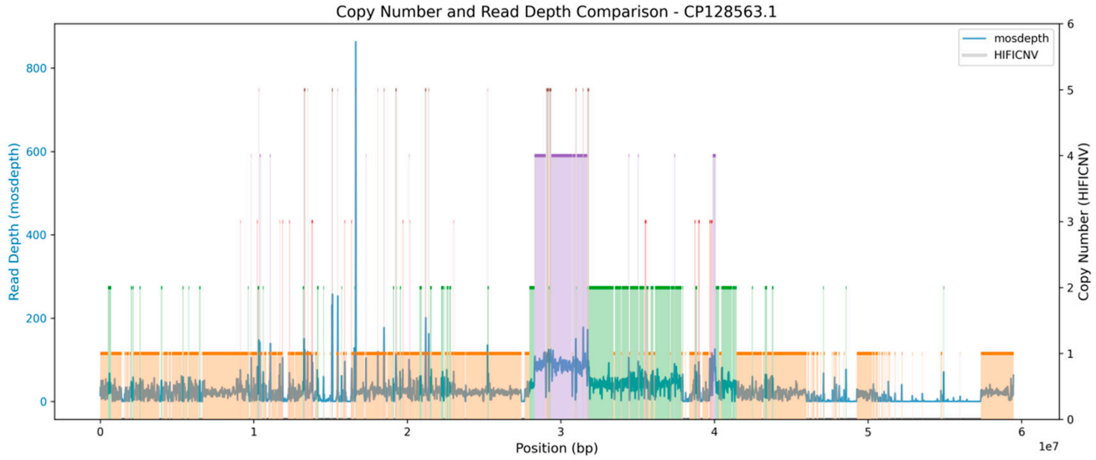


**Figure S15. Chromosome-wide CN estimation of Y assemblies.**

## References


1. Shumate, A.; Salzberg, S.L. Liftoff: accurate mapping of gene annotations. *Bioinformatics* **2021**, *37*, 1639–1643.
2. Tegenfeldt, F.; Kuznetsov, D.; Manni, M.; Berkeley, M.; Zdobnov, E.M.; Kriventseva, E.V. OrthoDB and BUSCO update: annotation of orthologs with wider sampling of genomes. *Nucleic Acids Res.* **2025**, *53*, D516–D522.
3. Boeckmann, B.; Bairoch, A.; Apweiler, R.; Blatter, M.-C.; Estreicher, A.; Gasteiger, E.; Martin, M.J.; Michoud, K.; O'Donovan, C.; Phan, I. The SWISS-PROT protein knowledgebase and its supplement TrEMBL in 2003. *Nucleic Acids Res.* **2003**, *31*, 365–370.
4. Olagunju, T.A.; Rosen, B.D.; Neibergs, H.L.; Becker, G.M.; Davenport, K.M.; Elsik, C.G.; Hadfield, T.S.; Koren, S.; Kuhn, K.L.; Rhie, A. Telomere-to-telomere assemblies of cattle and sheep Y-chromosomes uncover divergent structure and gene content. *Nat. Commun.* **2024**, *15*, 8277.
5. Smith, T.; Olagunju, T.; Rosen, B.; Neibergs, H.; Becker, G.; Davenport, K.; Elsik, C.; Hadfield, T.; Koren, S.; Kuhn, K. The first complete T2T Assemblies of Cattle and Sheep Y-Chromosomes uncover remarkable divergence in structure and gene content. *Research Square* **2024**.
6. Wan, R.-D.; Gao, X.; Wang, G.-W.; Wu, S.-X.; Yang, Q.-L.; Zhang, Y.-W.; Yang, Q.-E. Identification of candidate genes related to hybrid sterility by genomic structural variation and transcriptome analyses in cattle-yak. *J. Dairy Sci.* **2025**, *108*, 679–693.
7. Lou, Y.; Liu, W.; Wang, C.; Huang, L.; Jin, S.; Lin, Y.; Zheng, Y. Histological evaluation and Prdm9 expression level in the testis of sterile male cattle-yaks. *Livestock Science* **2014**, *160*, 208–213.
8. Wang, G. Morphological and cellular features of spermatogenesis in yak and cattle intergenetic hybrids. PhD Dissertation, University of Chinese Academy of Sciences, 2020.
9. Damm, E. A novel perspective on PRDM9-directed meiotic recombination: How interallelic interactions between meiotic regulator PRDM9 and X-chromosomal hybrid sterility locus HstX2 regulate hybrid fertility phenotypes. **2022**.
10. Dobzhansky, T. Genetics and the origin of species. **1951**.
11. Arter, M.; Keeney, S. Divergence and conservation of the meiotic recombination machinery. *Nat. Rev. Genet.* **2024**, *25*, 309–325.
12. Xu, J.; Li, T.; Kim, S.; Boekhout, M.; Keeney, S. Essential roles of the ANKRD31–REC114 interaction in meiotic recombination and mouse spermatogenesis. *Proc. Natl. Acad. Sci. U. S. A.* **2023**, *120*, e2310951120.
13. Qu, W.; Liu, C.; Xu, Y.-T.; Xu, Y.-M.; Luo, M.-C. The formation and repair of DNA double-strand breaks in mammalian meiosis. *Asian Journal of Andrology* **2021**, *23*, 572–579.
14. Acquaviva, L.; Boekhout, M.; Karasu, M.E.; Brick, K.; Pratto, F.; Li, T.; van Overbeek, M.; Kauppi, L.; Camerini-Otero, R.D.; Jasin, M. Ensuring meiotic DNA break formation in the mouse pseudoautosomal region. *Nature* **2020**, *582*, 426–431.
15. Stanzione, M.; Baumann, M.; Papanikos, F.; Dereli, I.; Lange, J.; Ramlal, A.; Tränkner, D.; Shibuya, H.; de Massy, B.; Watanabe, Y. Meiotic DNA break formation requires the unsynapsed chromosome axis-binding protein IHO1 (CCDC36) in mice. *Nat. Cell Biol.* **2016**, *18*, 1208–1220.
16. Robert, T.; Nore, A.; Brun, C.; Maffre, C.; Crimi, B.; Guichard, V.; Bourbon, H.-M.; De Massy, B. The TopoVIB-Like protein family is required for meiotic DNA double-strand break formation. *Science* **2016**, *351*, 943–949.
17. Brick, K.; Thibault-Sennett, S.; Smagulova, F.; Lam, K.-W.G.; Pu, Y.; Pratto, F.; Camerini-Otero, R.D.; Petukhova, G.V. Extensive sex differences at the initiation of genetic recombination. *Nature* **2018**, *561*, 338–342.

18. Xie, C.; Wang, W.; Tu, C.; Meng, L.; Lu, G.; Lin, G.; Lu, L.-Y.; Tan, Y.-Q. Meiotic recombination: insights into its mechanisms and its role in human reproduction with a special focus on non-obstructive azoospermia. *Hum. Reprod. Update* **2022**, *28*, 763–797.
19. Kauppi, L.; Barchi, M.; Baudat, F.; Romanienko, P.J.; Keeney, S.; Jasin, M. Distinct properties of the XY pseudoautosomal region crucial for male meiosis. *Science* **2011**, *331*, 916–920.
20. Rouyer, F.; Simmler, M.-C.; Johnsson, C.; Vergnaud, G.; Cooke, H.J.; Weissenbach, J. A gradient of sex linkage in the pseudoautosomal region of the human sex chromosomes. *Nature* **1986**, *319*, 291–295.
21. Lampitto, M.; Barchi, M. Recent advances in mechanisms ensuring the pairing, synapsis and segregation of XY chromosomes in mice and humans. *Cellular and Molecular Life Sciences* **2024**, *81*, 1–16.
